# Band-based similarity indices for gene expression classification and clustering

**DOI:** 10.1038/s41598-021-00678-9

**Published:** 2021-11-03

**Authors:** Aurora Torrente

**Affiliations:** Departamento de Matemáticas, Instituto Gregorio Millán, Universidad Carlos III de Madrid, 28911 Leganés, Spain

**Keywords:** Machine learning, Microarrays, Statistical methods

## Abstract

The concept of depth induces an ordering from centre outwards in multivariate data. Most depth definitions are unfeasible for dimensions larger than three or four, but the Modified Band Depth (MBD) is a notable exception that has proven to be a valuable tool in the analysis of high-dimensional gene expression data. This depth definition relates the centrality of each individual to its (partial) inclusion in all possible bands formed by elements of the data set. We assess (dis)similarity between pairs of observations by accounting for such bands and constructing binary matrices associated to each pair. From these, contingency tables are calculated and used to derive standard similarity indices. Our approach is computationally efficient and can be applied to bands formed by any number of observations from the data set. We have evaluated the performance of several band-based similarity indices with respect to that of other classical distances in standard classification and clustering tasks in a variety of simulated and real data sets. However, the use of the method is not restricted to these, the extension to other similarity coefficients being straightforward. Our experiments show the benefits of our technique, with some of the selected indices outperforming, among others, the Euclidean distance.

## Introduction

Quantification of gene expression data has become a benchmark in biomedical research for the study of numerous diseases. High-throughput techniques have been developed to monitor the level of expression of thousands of genes simultaneously across different samples. The potential heterogeneity of such samples is enormous, as they might include a variety of tissues of origin, cell types or diseases, as well as diverse time points or experimental conditions, such as stress, treatments or viral infections. This typically makes gene expression profiles very irregular and complex.

The nature of this type of data has raised tremendous statistical interest as it can be used to gain insight into human genetics by revealing hidden patterns or by providing accurate predicting models. Biological information about genes and samples can be incorporated into the data in the form of labels or “annotations” and used at some point of the analysis. If they are used from the beginning, the technique is said to be a supervised one, whereas in unsupervised approaches, they are ignored and just used to interpret the results. Many supervised and unsupervised methodologies have been tailored for the exploration of gene expression data^1–4^. Nevertheless, classical classification and clustering methods for their analysis are also widely extended, as they have permitted major advances such as identification of novel tumor subtypes^5–7^, prediction of gene functions^8,9^ or discrimination of patients with good or poor prognosis^10,11^. There are many studies that address the problem of identifying the most suitable technique for gene expression data among the plethora of possibilities (see, e.g.,^12–15^), which is, indeed, a difficult issue. But this is just the first step.

Most classification and clustering techniques have in common their need for quantifying pattern proximity, according to a certain criterion. The conventional approach consists in considering each gene as a point in an *m*-dimensional vector space, where *m* is the number of samples, or considering each sample as a point in an *n*-dimensional vector space, where *n* is the number of genes. In either case, this perspective allows using concepts and techniques from linear algebra for the analysis of gene expression data. In particular, the resemblance between two genes, for instance, can be stated in terms of a certain dissimilarity measure $\mathcal {D}$ between the respective vectors (or dually with a similarity measure $\mathcal {S}$ between them) in a metric space. A dissimilarity $\mathcal {D}({\mathbf {y}}_{i_1}, {\mathbf {y}}_{i_2})$ between vectors ${\mathbf {y}}_{i_1}$ and ${\mathbf {y}}_{i_2}$ in a sample $\{{\mathbf {y}}_{1}, \dots , {\mathbf {y}}_{n}\}$ is characterised by being non-negative: $\mathcal {D}({\mathbf {y}}_{i_1}, {\mathbf {y}}_{i_2})\ge 0$, symmetric: $\mathcal {D}({\mathbf {y}}_{i_1}, {\mathbf {y}}_{i_2}) = \mathcal {D}({\mathbf {y}}_{i_2}, {\mathbf {y}}_{i_1})$ and reflexive: $\mathcal {D}({\mathbf {y}}_{i},{\mathbf {y}}_{i})=0,\, \forall i=1,\dots ,n$. If in addition it satisfies the triangular inequality: $\mathcal {D}({\mathbf {y}}_{i_1}, {\mathbf {y}}_{i_2}) \le \mathcal {D}({\mathbf {y}}_{i_1}, {\mathbf {y}}_{i_3} ) + \mathcal {D}({\mathbf {y}}_{i_3}, {\mathbf {y}}_{i_2})$ $\forall i_3\in \{1,\dots ,n\}$, and is additively strict: $\mathcal {D}({\mathbf {y}}_{i_1}, {\mathbf {y}}_{i_2})=0 \iff {\mathbf {y}}_{i_1} = {\mathbf {y}}_{i_2}$, then it is called a metric. In general, classification and clustering algorithms can be used with any (dis)similarity measure. Yet, the selection of such a measure according to the specific problem is a pivotal decision to the quality of the analysis outcome, which has been traditionally overlooked (as put forward, for example, in^16–18^).

A well known example of dissimilarity is the Euclidean distance—indeed a metric—which is commonly used in every research discipline when the patterns to be compared have continuous features. However, this distance assumes a clean (noise-free) experimental vector space, which is an overly stringent premise for gene expression data. Another popular choice for quantitative data is the Pearson correlation distance, which has the drawback of presuming a linear relationship between similar vectors. These and many other distances in the literature (see, e.g.,^19^) commonly yield different results on the same expression data and the identification of the most suitable dissimilarity for a particular problem is still an open question.

On the other hand, other types of data, like qualitative or binary data, can be found in the gene expression context, with 1s representing expressed genes and 0s corresponding to not expressed ones, for instance. In this situation, there are better dissimilarity measures that account for their singular nature. In particular, numerous pairwise similarity indices have been proposed in the literature for binary data (e.g., refer to^20–24^). Though they are widely used in other applications, many of them have arisen in the ecology and geology fields for presence-absence data, where 1 designates a present attribute and 0 an absent one. There are several studies that compare similarity indices (see, for example^25–28^), but they commonly report different conclusions. This has motivated the general acceptance that the performance of similarity coefficients is also data-specific.

As a consequence, new ways of measuring proximity between pairs of objects (of diverse nature) have been further investigated in different contexts (e.g.,^29–33^).

In this work, we propose to construct a new (dis)similarity measure for multivariate quantitative observations by “transferring” quantitative information in the (gene or sample) vector space into a different space of binary matrices. This new space encodes the relative position of each original vector with respect to the other observations, which can be used to assess how similar profiles are.

Our methodology consists in first associating *d* binary vectors to each *d*-dimensional data point by borrowing certain elements from the finite-dimensional version of the Modified Band Depth (MBD)^34^: the bands defined by two or more distinct elements in the data set. The MBD is a particular definition of multivariate depth, a concept conceived to generalise univariate order statistics, ranks and medians to higher dimensions, that allows ordering multivariate data from center outwards.

A number of depth notions can be found in the literature (^35–37^ among others), but most of them have the drawback of being unfeasible for dimensions larger than 3 or 4. In contrast, the MBD is specially suitable for high dimensional observations and has been used in the DS and TAD methods^38^ to successfully classify gene expression data or in the BRIk algorithm^39^ to initialise k-means by making a smart use of the information conveyed by the data.

However, the ordering provided by this or alternative depth notions cannot be easily turned into a measure of proximity between multivariate points. Thus, addressing for instance the problem of clustering such observations by making use of some depth definition is not straight forward. As an exception to this, Jornsten^40^ proposed two depth-based methods, DDclust and DDclass, to efficiently cluster and classify, respectively, multivariate data, with a special application to gene expression. These are based on the $L_1$ depth^41^, which represents, for an observation ${\mathbf {y}}_i$ in a data set, the amount of probability mass needed at ${\mathbf {y}}_i$ to make this observation the multivariate median of the set.

Recently, Tupper et al.^33^ introduced a distance based on finding “informative” bands, defined by pairs of elements in the data set, and adapting the Jaccard index^20^ for set similarity. Such a distance was used to cluster non-stationary time series but was reported to be computationally expensive.

With a philosophy similar to that of^33^, but with a different and more general perspective, we propose to use bands defined by any number *j* of distinct elements –here referred to as *j*-bands–, to derive a similarity/dissimilarity measure for multivariate observations and to investigate its adequacy for gene expression data. In order to obtain these band-based indices we identify the position of each coordinate *k* of a data point ${\mathbf {y}}_i$ in a sample of *n* *d*-dimensional elements $\{{\mathbf {y}}_1,$ $\dots ,{\mathbf {y}}_n\}$, with respect to each possible *j*-band, and assign a bit to describe inclusion (1) or exclusion (0) inside it. In this way, each *d*-dimensional observation has an associated binary matrix of size ${n\atopwithdelims ()j}\times d$.

From the columns of these matrices, a collection of *d* contingency tables—one for each coordinate—can be computed for each pair of data points, and used to evaluate, coordinate by coordinate, how “resembling” they are, according to some conventional index. In our proposal, the computational cost of evaluating all ${n\atopwithdelims ()j}$ bands is bypassed by making use of a simple combinatorial strategy, as described in the Methods section. Also, we show that these—exponentially large—matrices do not need to be explicitly found or stored, as the corresponding contingency tables can be directly calculated from the observations.

Though there is a manifest interest in thoroughly examining the implications of choosing one of the many different similarity coefficients found in the literature in the analysis of gene expression data, here we just aim at illustrating the usefulness of the information summarised in the band-based matrices associated to each observation and its applicability to different techniques.

With this in mind, we propose to use—some—typical (dis)similarity measures for binary data as an alternative to classical distances for multivariate data in benchmark algorithms such as k Nearest Neighbours (kNN)^42^, k-means^43^ or Partitioning Around the Medoids (PAM)^44^, by identifying which ones are suitable for the analysis of gene expression data.

The rest of the manuscript is organised as follows. The Methods Section includes a detailed description of the proposed theoretical methodology and its efficient implementation to derive different (dis)similarity measures. The Results Section evaluates the use of such measures in classification and clustering tasks when compared to standard distances, for both synthetic and real data. The Discussion Section summarises the main findings and enhances the potential of the proposed band-based indices for the analysis of gene expression data. Final conclusions are reported in the last section.

## Methods

### Notation

**Figure 1:**
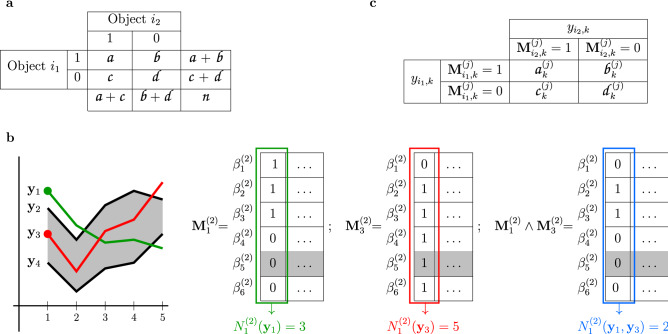
Computation of binary matrices. (**a**) Contingency table for binary vectors ${\mathbf {x}}_{i_1}$ and ${\mathbf {x}}_{i_2}$, associated to objects $i_1$ and $i_2$ respectively. (**b**) Representation of four quantitative observations with five variables that define six possible 2-bands, namely, $\beta _1^{(2)} = [{\mathbf {y}}_1; {\mathbf {y}}_2]$, $\beta _2^{(2)} = [{\mathbf {y}}_1; {\mathbf {y}}_3]$, $\beta _3^{(2)} = [{\mathbf {y}}_1; {\mathbf {y}}_4]$, $\beta _4^{(2)} = [{\mathbf {y}}_2; {\mathbf {y}}_3]$, $\beta _5^{(2)} = [{\mathbf {y}}_2; {\mathbf {y}}_4]$, $\beta _6^{(2)} = [{\mathbf {y}}_3; {\mathbf {y}}_4]$. The gray region between the black curves is the band $\beta _5^{(2)}$. The first coordinate of curve ${\mathbf {y}}_1$ (green dot) is outside the band; this corresponds to a 0 in the entry (5,1) of its associated Boolean matrix ${\mathbf {M}}_{1}^{(2)}$. The first coordinate of curve ${\mathbf {y}}_{3}$, in red, is inside the band; the entry (5,1) in matrix ${\mathbf {M}}_{{3}}^{(2)}$ is 1, accordingly. The corresponding Boolean product is 0, meaning that the band does not include both coordinates. (**c**) Binary contingency table for coordinate *k* and the number *j* of curves forming a band, constructed from the matrices sketched in (**b**).

In the context of binary data, given two objects $i_1$ and $i_2$, with respective associated binary vectors ${\mathbf {x}}_{i_1}$ and ${\mathbf {x}}_{i_2}$, the information required to compute a similarity index between ${\mathbf {x}}_{i_1}$ and ${\mathbf {x}}_{i_2}$—to describe the proximity of $i_1$ and $i_2$—can be summarised in a 2$\times 2$ contingency table (see Fig. 1a). With the usual notation, ${a}$ is the number of attributes present at both objects $i_1$ and $i_2$ (or equivalently, the number of binary features or bits equal to 1 in both ${\mathbf {x}}_{i_1}$ and ${\mathbf {x}}_{i_2}$); ${b}$ is the number of attributes present at object $i_1$ and absent at $i_2$; ${c}$ is the number of attributes absent at object $i_1$ and present at $i_2$; and ${d}$ is the number of attributes absent at both $i_1$ and $i_2$. Clearly, the sum ${n} = {a} + {b} + {c} + {d}$ is the total number of attributes, and the fractions ${p}_{i_1} = \dfrac{ {a}}{ {a} + {b}}$ and ${p}_{i_2}= \dfrac{ {a}}{ {a} + {c}}$ represent the proportion of attributes present at both objects $i_1$ and $i_2$, among the number of attributes present at $i_1$ and $i_2$, respectively. Note that in the context of information retrieval or machine learning these ratios correspond to the terms *precision* and *recall*.

On the other hand, given a sample of *n* *d*-dimensional observations $\{{\mathbf {y}}_1, \dots ,$ ${\mathbf {y}}_n\}$, where ${\mathbf {y}}_i=(y_{i,1},\ldots,y_{i,d})$, we define the *j*-band determined by the *j* distinct elements ${\mathbf {y}}_{i_1}, \dots , \mathbf {y}_{i_j}$, with $2\le j \le n$, as the *d*-dimensional interval delimited by the minimum and maximum of their coordinates, in the form $[{\mathbf {y}}_{i_1}; \dots ; {\mathbf {y}}_{i_j}]=\Big \{(y_{1},\ldots,y_{d}) \in \mathbb {R}^d :\min \{y_{i_{1}, k},\dots ,y_{i_{j}, k}\} \le y_{k}\le \max \{y_{i_{1}, k}, \dots ,y_{i_{j}, k}\},$ $k\in \{1,\dots ,d\}\Big \}$. However, for referring to *j*-bands in the cases where it is irrelevant to identify which elements define them, we simplify the notation by letting $B^{(j)}$ be the set of all *j*-bands $\beta _p^{(j)}$, $p=1,\dots , {n \atopwithdelims ()j}$: $B^{(j)}=\left\{ \beta ^{(j)}_1, \dots , \beta ^{(j)}_{n\atopwithdelims ()j}\right\}$.

Such *j*-bands are the basic elements for defining the MBD. This depth notion computes and averages the proportion of coordinates of element ${\mathbf {y}}_i$ lying within the limits of all possible *j*-bands, with $2\le j \le J$,   $2 \le J \le n$. More precisely, given $J\in \{2,3,\dots ,n\}$, the MBD for ${\mathbf {y}}_i$ is given by

1 $$\begin{aligned} MBD_{n,J}({\mathbf {y}}_i)=\sum _{j=2}^{J} MBD_n^{(j)}({\mathbf {y}}_i), \end{aligned}$$

where

2 $$\begin{aligned} MBD_n^{(j)}({\mathbf {y}}_i)=\displaystyle \frac{1}{d \times {{n}\atopwithdelims (){j}}} \sum _{1\le i_{1}<\dots <i_{j}\le n}\ \sum _{k=1}^{d} I_{\left\{ \min \left\{ y_{i_{1}, k},\dots ,y_{i_{j}, k}\right\} \le y_{i,k}\le \max \left\{ y_{i_{1}, k},\dots ,y_{i_{j}, k}\right\} \right\} } \ . \end{aligned}$$

For each *j*, we can associate to each ${\mathbf {y}}_i$ an ${n\atopwithdelims ()j}\times d$ binary matrix $\mathbf {M}_i^{(j)}$, whose (*p*, *k*) element equals 1 if $y_{i,k}$ lies within the *k*-th (one-dimensional) interval determined by the *p*-th *j*-band in $B^{(j)}$, and 0 otherwise. The number of 1s within the *k*-th column represents the number of bands in $B^{(j)}$ whose *k*-th interval contains $y_{i,k}$. Let us denote this number by $N^{(j)}_{k}({\mathbf {y}}_i)$.

In a similar way, given the pair of distinct elements $({\mathbf {y}}_{i_1}, {\mathbf {y}}_{i_2})$, we can construct the matrix $\mathbf {M}_{i_1,i_2}^{(j)}$ as the Boolean product of $\mathbf {M}_{i_1}^{(j)}$ and $\mathbf {M}_{i_2}^{(j)}$ to identify the bands in $B^{(j)}$ whose *k*-th interval contains the *k*-th coordinate of both points ${\mathbf {y}}_{i_1}$ and ${\mathbf {y}}_{i_2}$. The number of 1s in the *k*-th column of this matrix is denoted by $N^{(j)}_{k}({\mathbf {y}}_{i_1}, {\mathbf {y}}_{i_2})$.

### Similarity indices for binary data

Some of the most widely used similarity indices for binary data are described below.

- **Simple matching (SM) coefficient:** Introduced by^23^, it provides the simplest similarity measure as the proportion of matching binary attributes, both present and absent, of items $i_1$ and $i_2$:
  $$\begin{aligned} \mathcal {S}_{\text {SM}}(\mathbf {x}_{i_1}, \mathbf {x}_{i_2}) = \dfrac{ {a} + {d}}{ {a} + {b}+ {c} + {d}}. \end{aligned}$$
- **Jaccard (J) coefficient:** It was introduced in^20^ and is one of the most frequently used indices. In fact, the dissimilarity index derived from it is a metric. It counts the number of matching present attributes and compares it to the number of attributes that are present at least at one of the objects:
  $$\begin{aligned} \mathcal {S}_{\text {J}}(\mathbf {x}_{i_1}, \mathbf {x}_{i_2}) = \dfrac{ {a}}{ {a} + {b}+ {c}}. \end{aligned}$$
- **Simpson (S) coefficient:** This popular index was introduced in^22^ to measure set similarity as the proportion of the smallest set included in the largest one. It is often referred to as Lennon’s coefficient^45^, and corresponds to the largest of proportions ${p}_{i_1}$ and ${p}_{i_2}$:
  $$\begin{aligned} \mathcal {S}_{\text {S}}(\mathbf {x}_{i_1}, \mathbf {x}_{i_2}) = \dfrac{ {a}}{\min \{ {a} + {b} , \, {a}+ {c} \} }. \end{aligned}$$
- **Forbes (F) coefficient:** It was derived in the ecology field as the observed-to-expected ratio of the number of species in two random samples^21^. Despite its good general properties, this index has been routinely excluded from studies and discussions on similarity indices. Nevertheless, it has recently raised interest^28,46,47^ and it has been shown to outperform other indices in several situations. The corresponding formula is:
  $$\begin{aligned} \mathcal {S}_{\text {F}}(\mathbf {x}_{i_1}, \mathbf {x}_{i_2}) = \dfrac{ {a} \times ( {a} + {b}+ {c} + {d}) }{( {a} + {b} )\times ( {a}+ {c} ) }. \end{aligned}$$
- **Dice (D) coefficient:** Presented in^48^, and also known as S$\phi$rensen or Czekanowski coefficient, it is computed like the Jaccard index, but giving double weight to matching attributes with value 1:
  $$\begin{aligned} \mathcal {S}_{\text {D}}(\mathbf {x}_{i_1}, \mathbf {x}_{i_2}) = \dfrac{2 {a}}{2 {a} + {b}+ {c}}. \end{aligned}$$
- **Anderberg (A) coefficient:** It was used in^24^. It is another variation of the Jaccard coefficient that gives double weight to mismatching attributes:
  $$\begin{aligned} \mathcal {S}_{\text {A}}(\mathbf {x}_{i_1}, \mathbf {x}_{i_2}) = \dfrac{ {a}}{ {a} + 2( {b}+ {c})}. \end{aligned}$$
- **Ochiai (O) coefficient:** This index is described in^49^. It represents the geometric mean of ${p}_{i_1}$ and ${p}_{i_2}$. In several studies (see e.g.^27^) it has outperformed other indices. Its expression is:
  $$\begin{aligned} \mathcal {S}_{\text {O}}(\mathbf {x}_{i_1}, \mathbf {x}_{i_2}) = \dfrac{ {a}}{(( {a} + {b})( {a}+ {c}))^{1/2}}. \end{aligned}$$
- **Russell and Rao (RR) coefficient:** Introduced in^50^, it represents the proportion of positive matching attributes:
  $$\begin{aligned} \mathcal {S}_{\text {RR}}(\mathbf {x}_{i_1}, \mathbf {x}_{i_2}) = \dfrac{ {a}}{ {a} + {b}+ {c} + {d}}. \end{aligned}$$

All these coefficients are symmetric and non-negative similarity indices. When an index $\mathcal {S}$ with such properties lies in the interval [0, 1] it is possible to base on it the definition of a symmetric and non-negative index which measures dissimilarity as $\mathcal {D} = 1 - \mathcal {S}$. Note that this is not the case for F, which is not always bounded by 1; however, this issue can be circumvented by rescaling the values of F as in^28^. Also, note that $\mathcal {D}_{\text {RR}}=1 - \mathcal {S}_{\text {RR}}$ is not reflexive (i.e., not a proper dissimilarity) as $\mathcal {D}_{\text {RR}}(\mathbf {x}_i,\mathbf {x}_i)$ is not 0 for vectors $\mathbf {x}_i$ not identically equal to 1. Thus it is easy to anticipate that, normally, it will not produce good results.

### Proposed band-based similarity measure for quantitative data

Given the multivariate, quantitative data set under study $\{{\mathbf {y}}_1, \dots ,$ ${\mathbf {y}}_n\}$ and the number *j* used to define the bands, we can associate to each data point ${\mathbf {y}}_i$ a binary matrix $\mathbf {M}_i^{(j)}$, as previously explained. Figure 1(b) exemplifies the computation of these matrices through a toy data set with four 5-dimensional observations. These vectors define six different 2-bands, labelled $\beta _1^{(2)}, \dots , \beta _6^{(2)}$, where $\beta _1^{(2)}$ is the region determined by ${\mathbf {y}}_1$ and ${\mathbf {y}}_2$; $\beta _2^{(2)}$ is delimited by ${\mathbf {y}}_1$ and ${\mathbf {y}}_3$, and so on. We focus on elements ${\mathbf {y}}_1$, in green, and ${\mathbf {y}}_3$, in red, and examine their first coordinate: these are outside and inside, respectively, the band $\beta _{5}^{(2)} = [{\mathbf {y}}_2; {\mathbf {y}}_4]$, defined by the other two elements (gray region). Accordingly, the corresponding values in matrices $\mathbf {M}_{1}^{(2)}$ and $\mathbf {M}_{3}^{(2)}$ are 0 and 1, respectively, as highlighted in gray in Fig. 1(b). If we consider all other bands, we find that three of them contain the coordinate $y_{1,1}$ and five bands contain $y_{3,1}$; however, only two bands contain both coordinates simultaneously.

The key idea behind our method is the following. We expect that two observations whose coordinates are simultaneously embedded in many bands are more similar than those with coordinates located in few bands at the same time. Note that such similarity is dependent on the data set, i.e., two given elements will have different similarities when the other elements in the set change, in the same way the depth of a point is modified when the data set is altered.

Next, for each coordinate *k* and for each *j*, with $2\le j \le J$,   $2 \le J \le n$ (following the construction of the MBD), we can build a contingency table as the one shown in Fig. 1(c), where

$$\begin{aligned}& {a}_k^{(j)} = N_k^{(j)}(\mathbf {y_{i_1}}, \mathbf {y_{i_2}}),\\&\quad {b}_k^{(j)}=N_k^{(j)}( \mathbf {y_{i_1}}) - N_k^{(j)}(\mathbf {y_{i_1}}, \mathbf {y_{i_2}}),\\&\quad {c}_k^{(j)}=N_k^{(j)}( \mathbf {y_{i_2}}) - N_k^{(j)}(\mathbf {y_{i_1}}, \mathbf {y_{i_2}}) \end{aligned}$$

and

$$\begin{aligned} {d}_k^{(j)} = {n\atopwithdelims ()j} + N_k^{(j)}(\mathbf {y_{i_1}}, \mathbf {y_{i_2}}) - N_k^{(j)}( \mathbf {y_{i_1}}) - N_k^{(j)} ( \mathbf {y_{i_2}}) = {n\atopwithdelims ()j} - \left( {a}_k^{(j)} + {b}_k^{(j)} + {c}_k^{(j)}\right) .\end{aligned}$$

In our toy example, ${a}_k^{(2)} =2$, ${b}_k^{(2)} =1$, ${c}_k^{(2)} =3$ and ${d}_k^{(2)} =0$.

At this point, for a given *j*, it is possible to summarise the information contained in these *d* tables in two different manners: a) by adding corresponding values in all the tables and plugging the results in the chosen index, or b) by computing the chosen index component-wise and averaging across coordinates. To keep track of similarities between ${\mathbf {y}}_{i_1}$ and ${\mathbf {y}}_{i_2}$, it is more informative to use the second approach, as confirmed experimentally. Therefore, we used these counts to compute, coordinate by coordinate, the indices described in the previous subsection.

Finally, for a given *J*, if we denote by $\mathcal {S}_{k,j}$ the selected index computed for the pair (*k*, *j*), the band-based similarity measure that we proposed is simply the average of the coordinate-wise indices across the different values of *j*:

$$\begin{aligned} \mathcal {S} = \sum _{j=2}^J \sum _{k=1}^d \dfrac{\mathcal {S}_{k,j}}{d(J-1)} \, .\end{aligned}$$

Note that this similarity index is related to shape rather than to spatial proximity, which is appropriate for gene expression data. Note as well that each coefficient built in this way is obviously affine invariant and provides a sample-dependent similarity measure, making them suitable for data-driven tasks such as classification or clustering.

### Efficient computation of MBD and contingency tables

A direct implementation of expressions (1) and (2) to compute the MBD leads to nested for loops that become time-consuming as *J* increases. To overcome this problem, current applications of the MBD^51,52^ use the value $J=2$ because it is computationally faster and also because the order induced in the sample is very stable in *J*^34^. However, the effective construction of the ${n \atopwithdelims ()j}$ *j*-bands formed by *j* distinct observations from the sample becomes intensive as *n* increases, even for $j=2$.

To speed up computations, Torrente et al.^53^ provided an implementation of $MBD_{n,2}$ that avoids nested loops. Instead of exhaustively searching for all pairs of points in the sample, the alternative calculations are based on storing the data in an $n \times d$ matrix ${\mathbf {Y}}$ and increasingly ordering each column. Then

$$\begin{aligned} MBD_{n,2}({\mathbf {y}}_i)= \displaystyle \frac{1}{d \times {n \atopwithdelims ()2}} \displaystyle \sum _{k=1}^d \left( (n-l_k + 1) (l_k - 1 + \eta _k) - \eta _k^2 + {\eta _k \atopwithdelims ()2} \right) , \end{aligned}$$

where $\eta _k = \eta _{k,\mathbf {y_i}}$ is the multiplicity of $y_{i,k}$ within the *k*-th column of $\mathrm {\mathbf {Y}}$, and $l_k = l_{k,\mathbf {y_i}}$ is the first occurrence of $y_{i,k}$ in the same column of the reordered matrix. Also, we assume that $\displaystyle {\eta _k \atopwithdelims ()2} =0$ whenever $\eta _k=1.$

For a fixed *k* with $1\le k \le d$, a simple combinatorial strategy allows counting the number of pairs of elements from the sample such that the *k*-th coordinate of ${\mathbf {y}}_i$ is inside the interval defined by the *k*-th coordinates of those pairs. The complement principle states that

$$\begin{aligned} N^{(2)}_{k}({\mathbf {y}}_i) = \sum _{1\le i_{1}< i_{2}\le n} I_{\left\{ \min \left\{ y_{i_{1}, k},y_{i_{2}, k}\right\} \le y_{i,k}\le \max \left\{ y_{i_{1}, k},y_{i_{2}, k}\right\} \right\} } = {n \atopwithdelims ()2} - {l_k-1 \atopwithdelims ()2} - {n-l_k-\eta _k+1 \atopwithdelims ()2}, \end{aligned}$$

where again we assume $\displaystyle {\alpha \atopwithdelims ()2}=0$ whenever $0\le \alpha <2$.

This principle can also be used to count the number of *j*-tuples from the sample such that the *k*-th coordinate of ${\mathbf {y}}_i$ lies within the minimum and the maximum of the *k*-th coordinates of the *j* elements:

$$\begin{aligned} N^{(j)}_{k}({\mathbf {y}}_i)= \sum _{1\le i_{1}<\dots <i_{j}\le n} I_{\left\{ \min \left\{ y_{i_{1}, k}, \dots ,y_{i_{j}, k}\right\} \le y_{i,k}\le \max \left\{ y_{i_{1}, k}, \dots ,y_{i_{j}, k}\right\} \right\} } = {n \atopwithdelims ()j} - {l_k-1 \atopwithdelims ()j} - {n-l_k-\eta _k+1 \atopwithdelims ()j}, \end{aligned}$$

with $\displaystyle {\alpha \atopwithdelims ()j}=0$ whenever $0\le \alpha <j$. Thus, the MBD for $J>2$ can be computed, with the computational cost linearly—instead of exponentially—scaling with *J*, as

$$\begin{aligned} MBD_{n,J}({\mathbf {y}}_i)= \sum _{j=2}^{J} MBD_n^{(j)}({\mathbf {y}}_i) = \sum _{j=2}^{J} \dfrac{1}{d\times {n\atopwithdelims ()j}} \sum _{k=1}^d N^{(j)}_{k}({\mathbf {y}}_i) . \end{aligned}$$

Finally, given two points from the sample, ${\mathbf {y}}_{i_1}$ and ${\mathbf {y}}_{i_2}$, we make use of the principle of inclusion-exclusion to compute the number of *j*-tuples containing the *k*-th coordinate of both points. Thus we write

$$\begin{aligned}&N^{(j)}_{k}({\mathbf {y}}_{i_1},{\mathbf {y}}_{i_2}) = \sum _{1\le i_{1}<\dots <i_{j}\le n} I_{\left\{ \min \left\{ y_{i_{1}, k}, \dots ,y_{i_{j}, k}\right\} \le y_{i_1,k}, y_{i_2,k}\le \max \left\{ y_{i_{1}, k}, \dots ,y_{i_{j}, k}\right\} \right\} } =\\&\quad {n \atopwithdelims ()j} - {l_{k,M}-1 \atopwithdelims ()j} - {n-l_{k,m}-\eta _{k,m}+1 \atopwithdelims ()j} + {l_{k,M}- l_{k,m}- \eta _{k,m} \atopwithdelims ()j} ,\quad \text { if } m\ne M, \end{aligned}$$

and

$$\begin{aligned} N^{(j)}_{k}({\mathbf {y}}_{i_1},{\mathbf {y}}_{i_2}) = {n \atopwithdelims ()j} - {l_{k,M}-1 \atopwithdelims ()j} - {n-l_{k,m}-\eta _{k,m}+1 \atopwithdelims ()j} ,\ \text { if } m= M, \end{aligned}$$

where $$m=\min \{y_{i_1,k}, y_{i_2,k}\}$$ and $$M=max\{y_{i_1,k}, y_{i_2,k}\}$$.

**Figure 2:**
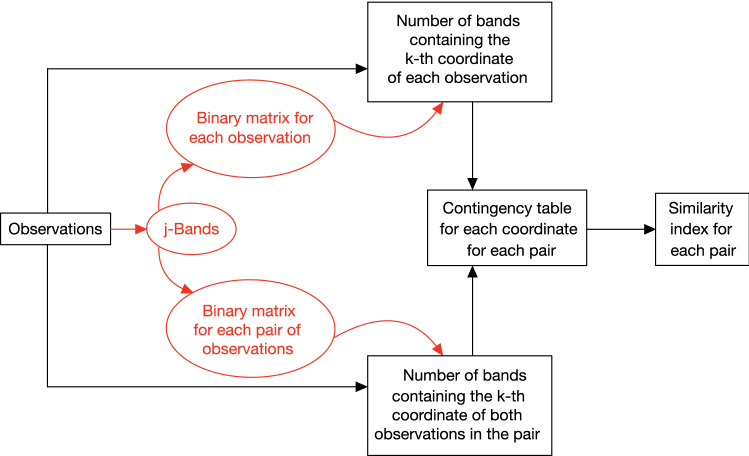
Method workflow. Calculations relative to red blocks can be skipped to alleviate the computational cost.

These calculations reveal the shortcut in the method workflow displayed in Fig. 2 (as black blocks): instead of computing each *j*-band and each binary matrix (red blocks), we can obtain the numbers $N^{(j)}_{k}(\cdot )$ and $N^{(j)}_{k}(\cdot ,\cdot )$ directly from the observations.

### Application to classification and clustering tasks

Finally, we describe how to examine the efficiency of the proposed dissimilarities indices in different standard techniques to be run on either simulated models or real data (see the Results section).

A straight forward application of the similarity indices to classification is given through the well known *k* Nearest Neighbours (kNN) algorithm^42^, a simple, widely-used rule that has proven to be very powerful. Though the Euclidean distance is the dissimilarity measure most frequently used with this method we also assess it when using other classical distances, namely: the Manhattan distance, the Minkowski distance, for $p\in \{0.25, 0.5,$ $0.75, 3, 4, 5\}$, and Pearson’s correlation-based distance. The assessment of how each of the selected dissimilarity measures affects the algorithm’s performance is carried out in terms of the classification error rates (i.e., the percentage of observations the method misclassifies). To have a common value for all measures, the number *k* of nearest neighbours used in the simulations was selected by the rule of thumb of setting *k* equal to the largest odd integer smaller than or equal to the square root of the number of training samples. The only exception to this is considered in one of the real datasets, where two of the classes are very small and the selection of *k* according to such a rule resulted in high classification error rates. Ties were broken by choosing the group containing the neighbour closest to the sample to be classified (that is, as if $k=1$).

For artificial data, we generated $B_a=200$ pairs of independent training and test sets, and used the test sets to evaluate the classification error. For real data, we estimated this error with 10-fold internal cross-validation $B_r=200$ times (see^54^).

Additionally, we have selected PAM, a deterministic method, to illustrate the performance of the dissimilarities indices in clustering artificial data with respect to the distances mentioned above. For each dissimilarity measure, the partitions produced by the algorithm were compared to the true ones using the clustering error rate described in^1^; that is, for each partition, we permuted the cluster labels and selected the minimum proportion of samples with labels disagreeing with the true ones. In addition to this, we computed the adjusted Rand index (ARI)^55^ to assess the quality of the clustering output by comparing the labels provided by the PAM algorithm to the real ones.

For real data, we considered complete-linkage hierarchical clustering instead, as the biological results yielded by this method are easier to visualise and interpret.

All these methods were used as implemented in the statistical R environment (http://www.R-project.org), in packages class, cluster and stats, respectively.

## Results

### Simulation study

For the simulated data, we considered normally distributed models with different dimension and number of groups, as detailed in Table 1.

**Table 1:** Description of the model parameters. The matrix $\mathbf {I}_n$ is the identity of dimension *n*. The matrix $\mathbf {A}_{i;n}$ is an $n\times n$ matrix with all entries equal to zero, except the *i*-th position in the main diagonal, which equals 1. Models 1 and 2 consist of two Gaussian clusters; models 3–5 are Gaussian mixture models with fixed levels of overlap.

Model	Dimension	Means	Covariance Matrices
Model 1 $G = 2$	100	$\varvec{\mu }_1 =\mathbf {0}_{100}$	$\varvec{\Sigma }_1 = \varvec{\Sigma _2} = \mathbf {I}_{100}$
		$$\varvec{\mu }_2 =\mathbf {0.5}_{100}$$	
Model 2 $G=2$	10	$\varvec{\mu }_1 =\mathbf {0}_{10}$	$\varvec{\Sigma }_1=\varvec{\Sigma }_2=\sigma \mathbf {I}_{10} + \delta \mathbf {A}_{6;10}; \, \sigma = 0.1; \, \delta \in \{0,1,2\}$
		$$\varvec{\mu }_2 =(1,0.8,\dots , 0.2,\mathbf {0}_5)$$	

MixSim	G	Dimension	Average Overlap
Model 3	3	15	0.01
Model 4	4	25	0.005
Model 5	5	35	0.002

#### Application to classification

We evaluated the performance of kNN with the band-based similarity indices with respect to that resulting from the use of the standard distances. However, prior to such a comparison, we carried out an analysis of the influence of the value of *J* on the classification performance. To that end, we considered $J\in \{2,3,\dots ,10\}$ for the models proposed.

For each of these models, we generated 200 training sets with 100 observations in each group, and 200 independent test sets with 25 elements in each class. We computed the classification error rate in each test set.

Supplementary Figure S1 shows the average classification error rate for each model along with the corresponding standard errors. This evinces the stability of all the methods (except for RR) as *J* increases, in agreement with the ordering induced by the MBD in the data, for different values of *J*^34^. This is also true even for values of *J* that have not been previously reported in the literature. Also, we can establish a rather persistent ranking of these indices for the selected models. Clearly, S is the best option, followed by O, whereas RR and A stand on the opposite side. However, there is a slight trend to deteriorate the performance, which is more remarkable for some indices than for others. According to these results, hereinafter in the comparison with classical distances we will report only results corresponding to $J=2$ and 3, which are the ones suggested to be used in practical situations.

**Figure 3:**
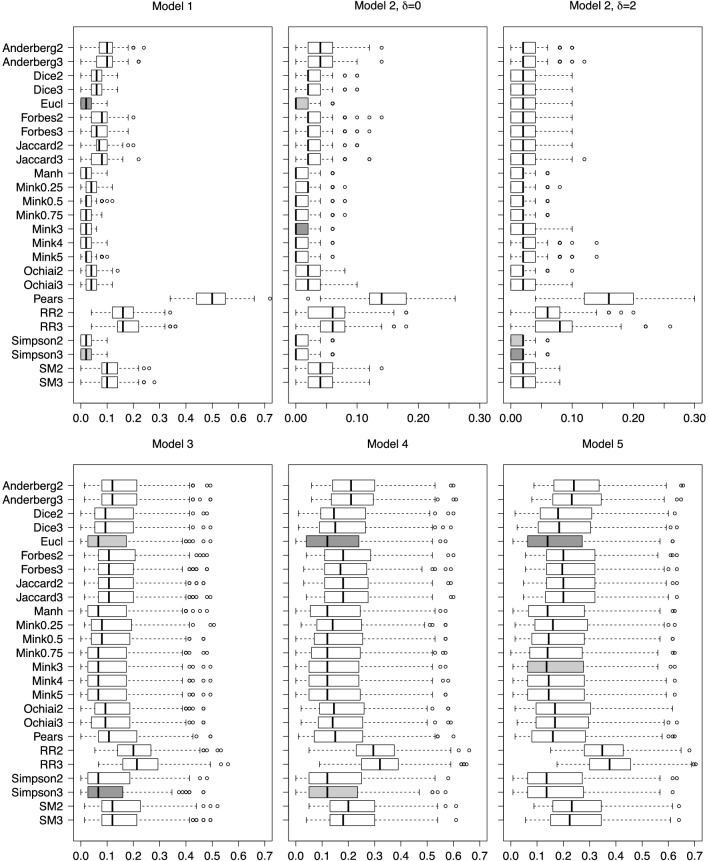
Classification error rates in the simulated models. Distribution of the classification error rates over 200 test sets from Models 1–5 after running kNN, using the classical distances and the dissimilarity indices based on bands, for $J=2$ and 3. Best and second best means are indicated with dark and light gray boxes, respectively. The number of observations in each class of the train and test sets is 100 and 25, respectively.

**Model 1**: It is taken from^56^ to mimic simple gene expression data and consists of two overlapping groups of points with 100 independent variables, drawn from normal distributions $N(\mathbf {0}_{100}, \mathbf {I}_{100})$ and $N(\mathbf {0.5}_{100}, \mathbf {I}_{100})$, where $\varvec {\alpha} _s$ is a vector of *s* identical components $\alpha$ and $\mathbf {I}_{s}$ is the identity matrix of size $s\times s$.

Here, most of the methods have an appropriate behaviour; see Fig. 3, upper-left panel. The classification error rates of the Euclidean distance, S, the Manhattan distance and the Minkovski distance, with $p=0.75,3,4$, are similar and very low, with averages below 0.03. Only A, SM and RR have errors one order of magnitude higher, and the Pearson distance has drastically bad results.

**Model 2**: It also has two groups, now with 10 independent variables; the last five ones are noise. The standard deviation of a single noise variable is increased an amount $\delta \in \{0,1,2\}$. The results corresponding to both extremes are displayed in Fig. 3, top-middle and top-right panels.

When $\delta =0$ the classical distances classify the new observations better than the band-based dissimilarity indices, but they are closely followed by S. If sorted from lowest to highest average error rates, S ranks 7 and 9 for $J=2$ and 3, respectively. Nevertheless, all the band-based measures except for RR yield average error rates lower than 0.05. Again, the Pearson correlation has the worst results. However, this model poses a critical scenario for the classical distances as the parameter $\delta$ rises. For $\delta =2$, the corresponding error rates deteriorate and S becomes the best option, whereas, notably, the band-based alternatives (except RR) remain robust against the value of $\delta$.

**Models 3–5**: These are models simulated by MixSim, a method proposed by^57^ that allows generating Gaussian mixture models with fixed levels of overlap between groups (in terms of probability). The respective number of groups are 3, 4 and 5; the respective number of variables are 15, 25 and 35 whereas the respective average overlaps are 0.01, 0.005 and 0.002.

These models are difficult scenarios for classification. The error rates are higher than in the previous cases and their distributions have larger interquartile ranges and contain many outliers (see the lower panels of Fig. 3) for all the methods. Despite that, the best techniques are again S and the Euclidean distance, followed by the Manhattan and Minkovski ($p=3,4$) distances.

#### Application to clustering

Next we evaluated the performance of the band-based indices for clustering methods. We generated 50 samples independently for each cluster, and simulated 200 data sets for Models 1–5. We applied PAM with all the band-derived measures and distances selected.

An inspection of the consistency of the results of each method across different values of *J* also shows they have a stable behaviour; Supplementary Figures S2 and S3 illustrate respectively clustering error rates and ARIs, again with a trend to deteriorate as *J* increases. As in the classification case, note that Supplementary Figs. S2 and S3 allow ranking the methods from best to worst consistently, regardless of the simulated model we consider.

With respect to the overall comparison, Tables 2 and 3 display clustering error rates and ARIs, averaged over the 200 simulated data sets, along with their standard errors (in brackets).

**Table 2:** Average clustering error rate over 200 simulations for all the simulated models; standard errors are written in brackets. Best and second best performance are highlighted in bold and italics, respectively.

Dissim.	M1	M2 ($\delta =0$)	M2 ($\delta =2$)	M3	M4	M5
A$_2$	0.3877 (0.0685)	0.2461 (0.0701)	0.2186 (0.0800)	0.4020 (0.0817)	0.5440 (0.0817)	0.6095 (0.0484)
A$_3$	0.3875 (0.0679)	0.2502 (0.0724)	0.2159 (0.0813)	0.4024 (0.0845)	0.5474 (0.0845)	0.6108 (0.0495)
D$_2$	0.3326 (0.0839)	0.1747 (0.0659)	0.1524 (0.0668)	0.2975 (0.0833)	0.4476 (0.0833)	0.5171 (0.0675)
D$_3$	0.3310 (0.0842)	0.1782 (0.0762)	0.1550 (0.0681)	0.2950 (0.0833)	0.4467 (0.0833)	0.5195 (0.0684)
Eucl	0.2072 (0.0821)	**0.0268** (0.0199)	0.3945 (0.0844)	0.1486 (0.0559)	0.2476 (0.0559)	0.3004 (0.0813)
F$_2$	0.3611 (0.0769)	0.2080 (0.0710)	0.1782 (0.0695)	0.3482 (0.0800)	0.4924 (0.0800)	0.5612 (0.0576)
F$_3$	0.3594 (0.0791)	0.2019 (0.0743)	0.1742 (0.0687)	0.3397 (0.0845)	0.4889 (0.0845)	0.5530 (0.0605)
J$_2$	0.3618 (0.0769)	0.2102 (0.0729)	0.1794 (0.0714)	0.3495 (0.0819)	0.4979 (0.0819)	0.5637 (0.0582)
J$_3$	0.3609 (0.0763)	0.2110 (0.0761)	0.1813 (0.0735)	0.3472 (0.0816)	0.4975 (0.0816)	0.5653 (0.0596)
Manh	0.2300 (0.0875)	0.0372 (0.0252)	0.1492 (0.1184)	0.1721 (0.0610)	0.2828 (0.0610)	0.3397 (0.0874)
Mk$_{0.25}$	0.3064 (0.0923)	0.1022 (0.0441)	0.1097 (0.0533)	0.2699 (0.0809)	0.4060 (0.0809)	0.4742 (0.0745)
Mk$_{0.5}$	0.2774 (0.0950)	0.0694 (0.0337)	0.0868 (0.0548)	0.2207 (0.0743)	0.3472 (0.0743)	0.4152 (0.0873)
Mk$_{0.75}$	0.2536 (0.0948)	0.0484 (0.0280)	0.0954 (0.0748)	0.1900 (0.0662)	0.3064 (0.0662)	0.3727 (0.0898)
Mk$_{3}$	0.2272 (0.0932)	*0.0290* (0.0212)	0.4356 (0.0479)	0.1556 (0.059)0	0.2650 (0.0590)	0.3231 (0.0802)
Mk$_4$	0.2566 (0.0918)	0.0324 (0.0239)	0.4436 (0.0439)	0.1659 (0.0579)	0.2948 (0.0579)	0.3508 (0.0821)
Mk$_5$	0.2816 (0.0854)	0.0363 (0.0250)	0.4492 (0.0375)	0.1795 (0.0619)	0.3171 (0.0619)	0.3846 (0.0824)
O$_2$	0.3132 (0.0866)	0.1592 (0.0650)	0.1404 (0.0625)	0.2753 (0.0821)	0.4153 (0.0821)	0.4828 (0.0733)
O$_3$	0.3124 (0.0888)	0.1578 (0.0702)	0.1433 (0.0677)	0.2752 (0.0819)	0.4159 (0.0819)	0.4862 (0.0754)
Pears	0.4630 (0.0282)	0.2374 (0.0329)	0.4340 (0.0464)	0.1311 (0.0489)	0.2323 (0.0489)	0.**2530** (0.0498)
RR$_2$	0.3998 (0.0620)	0.2640 (0.0889)	0.2456 (0.1030)	0.4491 (0.0877)	0.5738 (0.0877)	0.6358 (0.0459)
RR$_3$	0.4064 (0.0600)	0.2680 (0.0902)	0.2592 (0.1048)	0.4541 (0.0869)	0.5785 (0.0869)	0.6408 (0.0447)
S$_2$	*0.1838* (0.0768)	0.0645 (0.0440)	**0.0799** (0.0397)	*0.1268* (0.0518)	*0.2231* (0.0518)	0.2736 (0.0649)
S$_3$	**0.1795** (0.0744)	0.0598 (0.0461)	*0.0842* (0.0506)	**0.1234** (0.0550)	**0.2142** (0.0550)	*0.2615* (0.0634)
SM$_2$	0.3187 (0.0717)	0.1989 (0.0585)	0.1376 (0.0453)	0.2968 (0.0718)	0.4286 (0.0718)	0.5021 (0.0613)
SM$_3$	0.3498 (0.0706)	0.1988 (0.0543)	0.1342 (0.0470)	0.2975 (0.0715)	0.4273 (0.0715)	0.5128 (0.0625)

**Table 3:** Average ARI over 200 simulations for all the simulated models; standard errors are written in brackets. Best and second best performance are highlighted in bold and italics, respectively.

Dissim.	M1	M2 ($\delta =0$)	M2 ($\delta =2$)	MS1	MS2	MS3
A$_2$	0.0598 (0.0702)	0.2702 (0.1356)	0.3356 (0.1619)	0.1945 (0.0917)	0.1064 (0.0504)	0.0825 (0.0337)
A$_3$	0.0597 (0.0697)	0.2632 (0.1361)	0.3427 (0.1635)	0.1953 (0.0960)	0.1050 (0.0491)	0.0833 (0.0343)
D$_2$	0.1317 (0.1126)	0.4351 (0.1660)	0.4960 (0.1687)	0.3418 (0.1223)	0.2074 (0.0754)	0.1672 (0.0572)
D$_3$	0.1341 (0.1145)	0.4318 (0.1782)	0.4896 (0.1730)	0.3458 (0.1222)	0.2076 (0.0769)	0.1652 (0.0571)
Eucl	0.3636 (0.1722)	**0.8962** (0.0745)	0.0641 (0.1215)	0.6188 (0.1171)	0.4818 (0.1087)	0.4293 (0.1006)
F$_2$	0.0917 (0.0962)	0.3549 (0.1574)	0.4276 (0.1613)	0.2637 (0.1018)	0.1553 (0.0622)	0.1235 (0.0436)
F$_3$	0.0950 (0.1000)	0.3712 (0.1703)	0.4380 (0.1608)	0.2769 (0.1117)	0.1633 (0.0659)	0.1312 (0.0475)
J$_2$	0.0910 (0.0946)	0.3508 (0.1620)	0.4259 (0.1625)	0.2627 (0.1029)	0.1518 (0.0640)	0.1216 (0.0435)
J$_3$	0.0917 (0.0945)	0.3507 (0.1692)	0.4221 (0.1659)	0.2656 (0.1044)	0.1543 (0.0635)	0.1204 (0.0453)
Manh	0.3155 (0.1788)	0.8576 (0.0909)	0.5436 (0.2817)	0.5698 (0.1185)	0.4289 (0.1050)	0.3742 (0.1013)
Mk$_{0.25}$	0.1757 (0.1376)	0.6371 (0.1370)	0.6168 (0.1529)	0.3887 (0.1242)	0.2543 (0.0820)	0.2095 (0.0668)
Mk$_{0.5}$	0.2266 (0.1597)	0.7438 (0.1147)	0.6918 (0.1581)	0.4738 (0.1293)	0.3318 (0.0922)	0.2795 (0.0886)
Mk$_{0.75}$	0.2716 (0.1724)	0.8172 (0.1000)	0.6739 (0.2020)	0.5333 (0.1236)	0.3904 (0.0989)	0.3328 (0.0978)
Mk$_{3}$	0.3257 (0.1760)	*0.8882* (0.0792)	0.0165 (0.0353)	0.6043 (0.1192)	0.4539 (0.1066)	0.3963 (0.0956)
Mk$_{4}$	0.2632 (0.1582)	0.8754 (0.0878)	0.0110 (0.0281)	0.5804 (0.1153)	0.4070 (0.1000)	0.3563 (0.0946)
Mk$_{5}$	0.2121 (0.1341)	0.8612 (0.0913)	0.0064 (0.0214)	0.5526 (0.1203)	0.3714 (0.0949)	0.3135 (0.0877)
O$_2$	0.1613 (0.1283)	0.4761 (0.1700)	0.5283 (0.1649)	0.3773 (0.1244)	0.2437 (0.0793)	0.2031 (0.0675)
O$_3$	0.1641 (0.1346)	0.4828 (0.1757)	0.5225 (0.1771)	0.3782 (0.1239)	0.2444 (0.0834)	0.2014 (0.0693)
Pears	0.0014 (0.0143)	0.2745 (0.0701)	0.0163 (0.0336)	0.6568 (0.1090)	*0.5891* (0.0832)	*0.5746* (0.0814)
RR$_2$	0.0464 (0.0610)	0.2469 (0.1619)	0.2945 (0.1921)	0.1467 (0.0913)	0.0818 (0.0466)	0.0659 (0.0302)
RR$_3$	0.0404 (0.0567)	0.2406 (0.1620)	0.2690 (0.1902)	0.1419 (0.0900)	0.0794 (0.0477)	0.0630 (0.0304)
S$_2$	*0.4177* (0.1805)	0.7952 (0.1371)	**0.8093** (0.1258)	* 0.6771* (0.1072)	0.5841 (0.0982)	0.5604 (0.0864)
S$_3$	**0.4272** (0.1786)	0.8117 (0.1456)	*0.7989* (0.1480)	**0.6865** (0.1075)	**0.5989** (0.0943)	**0.5800** (0.0869)
SM$_2$	0.1433 (0.1030)	0.3699 (0.1326)	0.5286 (0.1272)	0.3370 (0.1040)	0.2206 (0.0671)	0.1778 (0.0507)
SM$_3$	0.1011 (0.0912)	0.3684 (0.1238)	0.5392 (0.1329)	0.3342 (0.1027)	0.2215 (0.0709)	0.1678 (0.0514)

The best similarity measures to cluster Model 1 are S and the Euclidean distance, followed by the Minkovski ($p=3$) and Manhattan ones. Pearson, RR, A, F and (surprisingly) J have the worst performance, with average clustering errors above 35% and average ARIs below 0.1.

For Model 2, the performance of the different methods resembles that they had in classification. The Euclidean distance, followed by the Manhattan and the Minkowski distances—for most of the reported values of *p*–, and then by S, are the best alternatives when $\delta =0$. However, when $\delta =2$, the Euclidean and Minkowski (for $p\ge 3$) distances yield drastically high clustering error rates and low ARI values, as reflected in Tables 2 and 3; the Manhattan and Pearson distances also deteriorate remarkably their behaviour. However, S stays rather stable and appears as the best option. The other band-based indices improve their performance, but yet they are not capable of beating S.

With respect to Models 3-5, only the Pearson distance, amongst the classical distances, is a competitor for S, with ARI values slightly smaller than those of S ($J=3$) and a better clustering error rate in Model 5. The other classical distances follow these techniques, whereas the use of the other band-based indices, specially A and RR, is clearly discouraged.

Thus, in this context, the band-based dissimilarity index S reveals to be consistently competitive with the classical distances, specially the Euclidean one, outperforming it in some of the simulated scenarios. It also beats D, F, O and, notably, the popular Jaccard index, though these have a reasonable performance. On the other hand, RR, A and SM are not efficient in order to reflect similarities between samples.

### Real gene expression data

To explore the effectiveness of the band-based indices with gene expression data in both classification and clustering techniques, we analysed different publicly available data sets, from both microarrays and RNA-seq technologies.

#### Microarray data

All the raw data sets were preprocessed following the normalisation described in^2^.

In kNN, the number of genes considered in each iteration was reduced to 200 in a supervised manner, using the B/W criterion^58^. This feature selection was performed for each training set.

For the (unsupervised) clustering approach, we kept the 200 most variable genes and clustered the samples, for which we knew the group labels (i.e., the annotations). We do not report here the results corresponding to the use of PAM, because it produced poor results in all the cases when the number of clusters was set equal to the number of classes. This is a common situation in gene expression data when samples are clustered. Therefore, instead of PAM, we used hierarchical clustering in conjunction with heatmaps, as these can be visually examined.

**Lymphoma data set.** It is a subset of the set presented in^6^; it contains 62 samples from three of the most prevalent lymphoid malignancies: 42 diffuse large B-cell lymphomas (DLBCL), 9 follicular lymphomas (FL) and 11 chronic lymphocytic leukemias (CLL).

**Figure 4:**
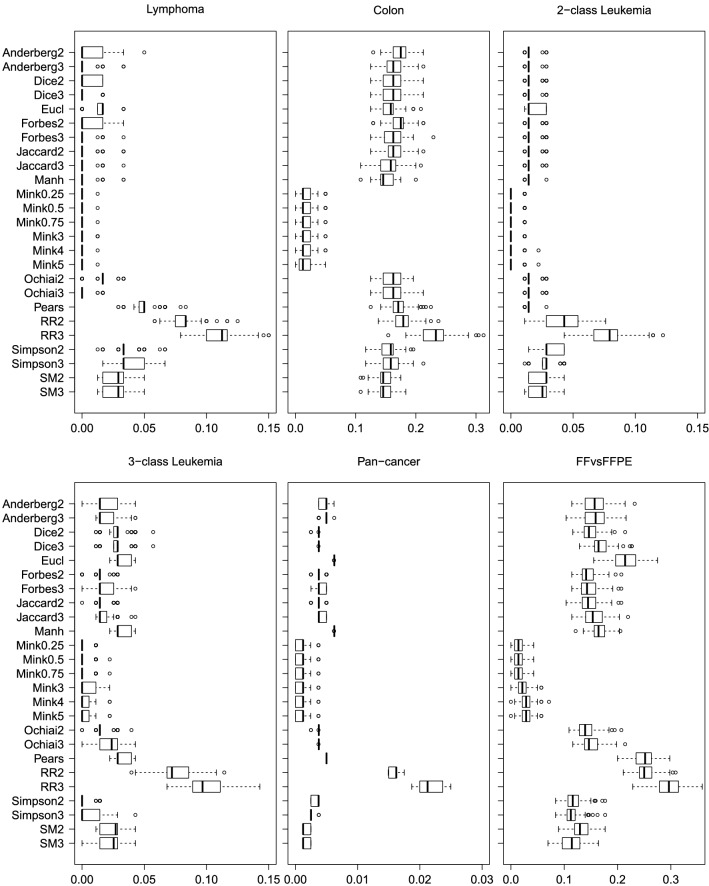
Classification error rates in real data sets. Distribution of kNN classification error rates for the lymphoma (top left), colon (top middle), leukemia with two (top right) and three classes (bottom left), RNAseq storage conditions (bottom middle) and pan-cancer (bottom right) data sets, using 10-folds cross-validation, with selection-bias correction.

Figure 4, top-left panel, displays the distribution of the 10-fold cross-validation classification errors made by kNN, that is, the proportion of wrongly classified elements, for the band-based indices and for the classical distances. A, D, J, all Minkovski distances and also F (when $J=2$) and O (when $J=3$) produce zero error rates in most of the iterations, being notably better than the classical Euclidean, Manhattan or Pearson distances. On the contrary, SM and—strikingly—S are slightly worse; RR misclassifies much more samples than the other methods.

On the other hand, after filtering the genes for clustering, we computed, for each pair of samples, the band-based dissimilarity measures, as well as the other classical distances. We used these to build a hierarchical tree using complete linkage.

**Figure 5:**
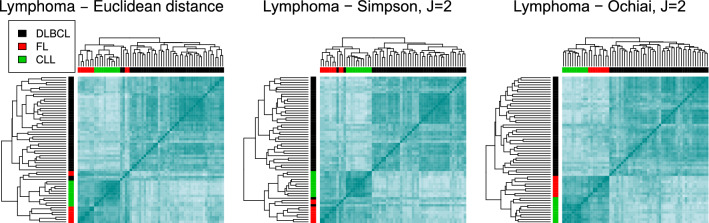
Clustering the lymphoma data set. Dendrograms and heatmaps for the lymphoma data set, using the Euclidean distance (left), the Simpson index (middle) and the Ochiai index (right), for $J=2$. The Euclidean distance merges 2 DLBCL and 2 FL samples with the CLL group. S identifies the structure better, misplacing 1 DLBCL in the FL group and 1 DLBCL in the CLL group. O completely restores the three classes.

We illustrate some of the results produced by the different indices in Fig. 5. In particular, we display the dendrograms and heatmaps corresponding to the Euclidean distance and to S and O, with $J=2$, as they are, in general, the best choices. The plots corresponding to the other measures are included in Fig. S4 from the Supplementary material.

The class labels are colour encoded. The Euclidean distance misplaces 2 DLBCL samples and 2 FL samples in a distinct branch of the dendrogram, which includes all the CLL ones. On the contrary, S separates the 3 groups for both values of *J*, but misplaces 2 DLBCL cells, one in each of the other groups. On the other hand, the band-based index O, as well as indices A ($J=3$), D and F, completely restore the three groups in three neat branches, and J and SM misplace 1 sample. The Minkovski distance, with $p=4$ and 5 works poorly, as it confounds FL and CLL groups. None of other classical distances behaves better than the band-based indices, where the number of wrongly allocated samples is between 1 and 5.

**Colon data set.** It consists of 2000 genes measured on 40 colon cancer patients and 22 healthy patients^59^.

With respect to classification, we report in Fig. 4, top-middle panel, the distribution of the classification error rates for the compared methods. Clearly, all Minkovski options provide the best results, with average error rates below 0.02, whereas RR with $J=3$ has the worst behaviour. The classical distances Manhattan, Euclidean and Pearson have a performance similar to that of most of the band-based indices, with average error rates ranging between 0.15 and 0.18, except for S, whose error rate is slightly below 0.15.

**Figure 6:**
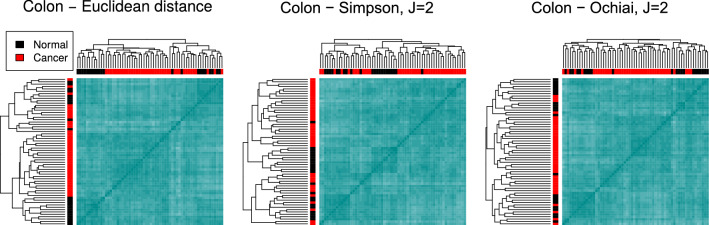
Clustering the colon data set. Dendrograms and heatmaps for the colon data set, using the Euclidean distance (left), the Simpson index (middle) and the Ochiai index (right), for $J=2$. The types of samples are difficult to separate and one branch with mixed leaves emerges in the three dendrograms.

Figure 6 displays the hierarchical clusterings using the Euclidean distance and the band-based indices S and O, with $J=2$. The Euclidean distance creates a dendrogram where the two main branches contain both normal and cancer samples; the leftmost branch contains two well separated sub-branches (normal and cancer) whereas the rightmost one mixes both types. The heatmap evinces that both groups are indeed difficult to tell apart. This is not surprising as gene expression in normal gastrointestinal tissues is known to be similar to that of neoplastic samples (see, for example^60^).

The use of S results in a similar situation, regardless of the value of *J*: both main branches in the tree contain normal and cancerous samples, but the second one contains two neater sub-branches, roughly corresponding to both classes. An analogous behaviour can be observed for most of the other methods, as illustrated in Fig. S5 of the Supplementary material. Nevertheless, SM and the Minkovski distance for $p=3$ reflect the similarities between observations in a more accurate way and find a clearer structure in the data according to the disease status. Most of the samples from each type are placed together in the dendrogram, forming two distinct branches, with only a few misclassified leaves.

**Leukemia data set.** This data set was made publicly available in^61^, and contains the gene expression levels in 72 leukemia samples, 25 of which were AML and 47 were ALL; additionally, 38 ALL samples originated from B-cells and 9 from T-cells. After the preprocessing steps, the expression matrix included 3571 genes and 72 samples. We first analysed the data considering two classes, AML and ALL, and next, also distinguishing the three groups.

The results corresponding to classification are illustrated in Fig. 4, top-right and bottom-left panels. The Minkovski distances have the best behaviour in kNN for the 2-class case. The Manhattan and the Pearson distances and also most of our band-based indices outperform the Euclidean distance; only S and SM (when $J=2$) are not better, in addition to RR, which, as before, is much worse. With respect to the 3-class case, the performance of S is drastically improved, reaching error rates comparable to or even better than those of the Minkovski options. Setting RR aside, the Euclidean, Manhattan and Pearson distances are the worst choices.

**Figure 7:**
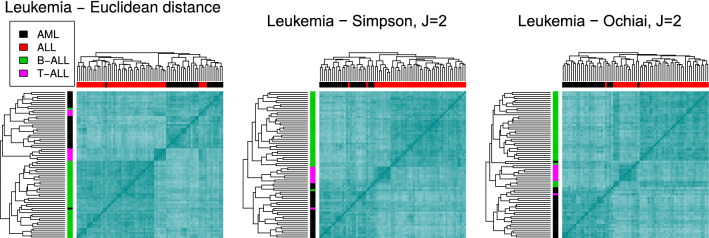
Clustering the leukemia data set. Dendrograms and heatmaps for the leukemia data set, using the Euclidean distance (left), the Simpson index (middle) and the Ochiai index (right), for $J=2$. The colour labels in the columns and the rows correspond to the 2-class and 3-class scenarios, respectively.

With respect to clustering, the top dendrogram in Fig. 7, left panel, shows that the Euclidean distance does not correctly restore the biological truth of the data, as it allocates some of the ALL samples (and in fact, all the T-ALL samples—see the hierarchical tree in the vertical axis) together with the AML samples. Also, the Euclidean distance misplaces one more AML sample in the dendrogram. However, the Simpson index finds the AML/ALL structure, wrongly assigning only 2 samples for $J=2$. Also, S identifies the subclusters containing T-cell ALL and B-cell ALL samples. O ($J=2$) also separates the three classes, though wrongly assigning 6 samples.

Results corresponding to alternative band-based indices and classical distances are shown in Fig. S6 of the Supplementary material, where we can see that most of the similarity measures used are capable of retrieving the 2-group structure. The exceptions are RR, SM and Minkovski with $p=5$, which mix both types of cells. However, only F and J and the Manhattan distance identify the three groups—with some wrongly allocated samples.

#### RNA-seq data

Next Generation Sequencing (NGS) provides more accurate data than microarrays, though their analysis is more challenging and complex. In this section, we additionally test the performance of the proposed indices with two NGS data sets.

**Pan-cancer data set.** This is a subset of the PANCAN data set^62^, downloaded from the UCR Time Series Classification Archive^63^. It contains the RNA-Seq gene expression levels of 20531 genes, measured by Illumina HiSeq platform and monitored in 801 tissue samples, randomly withdrawn from the original standardised dataset named the TCGA Pan-Cancer Clinical Data Resource (TCGA-CDR) set. Alignments were performed on the GRCh38 reference genome; read counts were measured on a gene level using HTSeq and normalised using the Fragments Per Kilobase of transcript per Million mapped reads (FPKM). The set comprises five types tumor: breast (BRCA), kidney (KIRC), colon (COAD), lung (LUAD) and prostate (PRAD) carcinomas. The groups are very distinct and easy to separate, as previously reported (see, for example^64^).

This leads to excellent classification results with the kNN algorithm. The Minkovski distances are capable of correctly classifying all the samples in 25% of the iterations, though their median (corresponding to 1 misclassified element) matches that of S. The other band-based indices, except for RR, have a slightly worse performance, but better than that of the Pearson, Manhattan and Euclidean distances.

**Figure 8:**
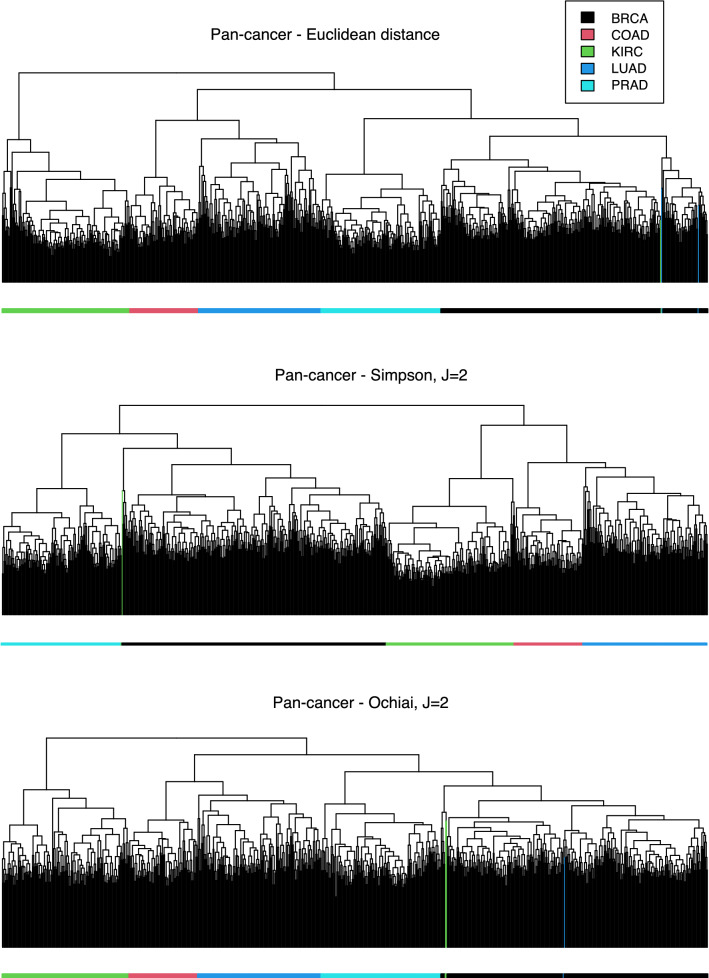
Clustering the Pan-cancer data set. Dendrograms for the Pan-cancer data set, using the Euclidean distance (top), the Simpson (middle) and the Ochiai (bottom) indices, with $J=2$. Wrongly assigned samples are coloured in the dendrogram.

On the other hand, due to the massive size of the data set, in Fig. 8 we only visualise, for clarity, the corresponding dendrograms, where wrongly allocated samples are highlighted. The good separability of the samples allows all the methods to identify the five types of cancer with high accuracy. The Euclidean distance places two kidney cancer and one lung cancer samples inside the the breast cancer branch, and identifies the kidney cancer type as the most distinct one (last merging of the dendrogram). A similar performance can be observed for O ($J=2$), with the same wrongly assigned KIRK and LUAD samples, but this time they are found to have some differences, as they are placed at different sub-branches of the BRCA branch. On the contrary, S ($J=2$) only assigns one of the KIRK samples to the BRCA branch, and finds KIRK to be more similar to COAD and LUAD.

None of the classical distances is capable of getting better results (see Fig. S7 of the Supplementary material). For most of them, the number of wrongly allocated samples ranges from 2 to 4, but the Minkovski distance with $p=0.25$ splits the LUAD samples into two distinct branches in the hierarchical tree. The other band-based indices have a similar performance, although the use of $J=3$ leads, in general, to slightly worse assignments, specially with the Jaccard index. Notably, RR and SM identify the correct structure, without any error for both values of *J*.

**FFvsFFPE data set.** This data set was downloaded from the ArrayExpress database^65^, under accession number E-MTAB-2523. The experiment included 86 RNA-seq samples representing six different human tissues: bladder carcinoma, prostate carcinoma, colon carcinoma, normal liver, normal colon and reactive tonsil^66^. These tissues were stored under two different conditions: formalin fixed, paraffin embedded (FFPE) and fresh frozen (FF), which affect nucleic acid extraction. The experimental design defines 9 known distinct classes indicated in Table 4, along with their size. The trimmed RNA-Seq reads were mapped against gene regions and transcripts were annotated using the RNA-Seq Analysis tool CLC Bio, version 6.0.1; the final processed files contained RPKM values for 19137 genes.

**Table 4:** FFvsFFPE data. Experimental design according to the tissue of origin and the storage condition.

Tissue type	Storage condition	Number of samples
Bladder carcinoma	FF	18
	FFPE	12
Colon carcinoma	FF	12
	FFPE	16
Normal colon	FF	6
Normal liver	FFPE	4
Prostate carcinoma	FF	7
	FFPE	7
Normal tonsil	FFPE	4

First, as mentioned before, if we stick to the given rule for selecting *k*, the classification error rates in kNN are remarkably higher than in previous data sets. This is because some of the groups are very small and do not have enough representatives to get the majority vote. In such a case, average error rates are in the order of 0.25. Therefore, we show in Fig. 4, bottom-right panel the results corresponding to $k=3$ for this dataset. As in previous data sets, the Minkovski distances have the best performance, followed by S and SM. The worse behaviour corresponds to the Euclidean and the Pearson distances and the RR index, whereas the other ones lie in between, with similar average error rates around 0.15.

**Figure 9:**
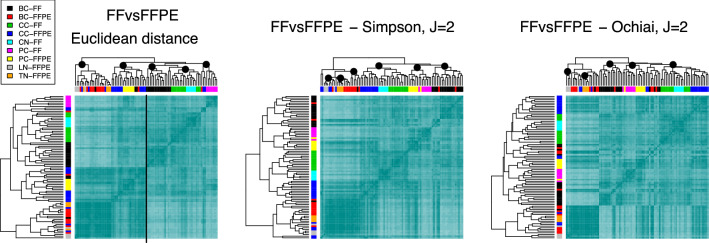
Clustering the FFvsFFPE data set. Dendrograms and heatmaps for the FFvsFFPE data set, using the Euclidean distance (left), the Simpson (middle) and the Ochiai (right) indices, with $J=2$. Main branches are highlighted with black dots.

Next, we built the heatmaps and hierarchical trees associated to the clustering procedure; see Fig. 9. The Euclidean distance leads to a heatmap with two differentiated parts, corresponding to FFPE and FF samples, respectively. These are indicated with a vertical black line; meaningful branches, identified by visual inspection, are highlighted with black dots. Inside the FFPE block, which includes some FF bladder carcinoma samples, we can identify a branch with all liver and tonsil samples and most FFPE bladder cancer samples; a neat difference between these cell types is not found, though. All FFPE prostate and most FFPE colon tumours are clustered together in another branch. On the FF side, the samples are correctly arranged according to the tissue of origin, except for a few FFPE samples. Cancer and normal colon tissues are placed in the same branch, as expected^60^.

Interestingly, the S and O indices, shown in the middle and right panels of Fig. 9, produce a different arrangement. To the left of the tree we find a branch with tonsil samples, grouped in a more consistent manner than with the Euclidean distance; also most liver samples are closely located in the tree. There is a branch containing all prostate cancer samples, subclassified according to the storage condition, in contrast to the previous result. Another sub-branch contains colon samples, regardless of the disease condition. S separates both types of bladder samples in distinct sub-branches, whereas O finds a group with mostly FF samples, while spreading the FFPE ones along the hierarchical tree.

Figure S8 in the Supplementary material shows the behaviour of the other techniques. None of them is capable of producing a neat separation between the classes. All the classical distances yield to inaccurate branches, except for Minkovski with $p=0.25$ and 0.5, which identify most of the tissues of origin but fail to retrieve the FFPE bladder carcinoma branch. On the other hand, most of the band-based similarities produce good results as they are also able to identify differences involving tissues of origin. This evinces that in this data set the band-based indices find, in general, more biologically meaningful similarities than the classical distances.

It is worth noting that since the class labels are known, it is possible to filter the genes using the B/W criterion also for clustering, but using the most variable ones suits the unsupervised philosophy better. Nevertheless, we repeated the analyses selecting the genes with that criterion. The results, in terms of structure recovery, were very similar to the ones previously discussed.

In summary, we conclude from the previous results that, even though not all the dissimilarity coefficients based on bands have an appropriate behaviour for every (simulated) dataset, when it comes to efficiently assessing biological differences between samples they reveal as suitable alternatives, especially in clustering tasks. In particular, the Simpson and the Ochiai indices are, globally, the best options: they have consistently good or the best performance across our variety of experiments.

## Discussion

Our findings from the Results section suggest that the proposed technique for constructing a similarity or dissimilarity measure for quantitative data is effective for analysing gene expression data. However, it is clear that there are always scenarios where a particular dissimilarity/distance measure is not the best option, as it has been illustrated in this study.

We have first evaluated the performance of these indices in relation to *J*, the largest number of different observations considered to form the *j*-bands. This is computationally feasible due to the efficiency of the proposed method. Our experiments show, as expected, that the results produced for different values of *J* are very similar, but sometimes with a tendency to deteriorate as *J* increases, a fact that has not been previously reported. Therefore, it is enough to set *J* equal to 2 or 3 to get satisfactory results fast. We can rank the band-based dissimilarity indices that we have considered from best to worst and identify which of them have systematically a bad behaviour (or worse than the competitors) in the analysed simulated data sets, i.e., with distributions that are spherical or with ellipsoidal weighted components. These indices are the RR and A measures, which seem less suitable for classifying or clustering this kind of data; this is consistent to what has been reported in several studies with respect to RR. Surprisingly, despite being one of the most popular indices, J does not perform as well as expected. Instead, S is clearly the best option in most of the tested data sets, as it achieves lower classification/clustering error rates and higher ARIs than every other dissimilarity measure, including, for instance, the benchmark Euclidean distance.

In the case of analysing gene expression data, the differences among the band-based methods are much smaller as most of them retrieve the underlying biological structure. The variety of analised data sets illustrates several situations where some of the band-based indices outperform the classical distances, especially for clustering tasks, earning their right to be considered a suitable measure of similarity in the gene expression context. As an example, the ability to cluster together samples from the same tissue of origin but stored under different conditions, or to better arrange samples according to the disease status, appears to be of particular interest. As opposed to the simulated Gaussian data case, the Jaccard index has a more relevant behaviour in this context.

## Conclusions

Different applications such as pattern recognition or classification of multivariate data require a similarity measure $\mathcal {S}$ or dissimilarity measure $\mathcal {D}$. The choice of such an appropriate measure relies on the data to be analysed, and in particular on the nature of their variables. Thus, for example, the Euclidean distance is a common measure for continuous data whilst binary data is often examined with indices such as Jaccard’s or Simpson’s ones.

In this work we propose a similarity/dissimilarity measure for continuous data based on binary features associated to the original observations. To that end, we make use of the idea underlying in the construction of the MBD, a data depth notion that has proven useful and efficient in the analysis of complex data, like gene expression data. More precisely, for each coordinate of a given observation we build a binary vector that helps count how many bands in the sample contain such coordinate. These vectors allow constructing contingency tables as in the presence-absence context, which become the input of standard similarity indices. The computation of the contingency tables is very efficient as it is possible to determine the corresponding values without evaluating each band. Instead, we use a combinatorial approach after reordering the columns of the data in an increasing way.

We have used this strategy for analysing a collection of simulated and real gene expression data sets using supervised and unsupervised classification. In summary, it is apparent that the binary matrices constructed from the bands keep track of similarities appropriately, though not all indices make a sound use of this information in every scenario. For simulated data sets, with (Gaussian) spherical or with ellipsoidal weighted-component distributions, the Simpson index is the best choice, followed by the Ochiai index. On the contrary, for more complex real gene expression data, additional band-based indices (e.g., the Dice index, the Forbes index—in agreement with recent work—or the popular Jaccard index) come to the fore as better alternatives than the widely-used Euclidean, Manhattan or Pearson distances. In some cases, S and O are not the best options, yet their performance is good enough to be considered as relevant choices. The different Minkovski distances are the most appropriate ones for classification tasks in these data sets, but are outperformed by S and O in clustering. In brief, S and O are sound options that produce competitive, sometimes complementary, outputs. Therefore, a joint analysis using them is suggested.

In either case, the technique presented in this study to construct a (dis)similarity measure based on bands from the MBD notion reveals as a suitable alternative, which can be extended to any of the multiple similarity indices defined for binary data and thus applied to quantitative data.

## Supplementary Information

Supplementary Information.
