# Supplementary material for: Band-based similarity indices for gene expression classification and clustering
Source: Sci Rep. 2021 Nov 3;11:21609. doi: 10.1038/s41598-021-00678-9 (PMC8566472; doi:10.1038/s41598-021-00678-9)

Supplementary Material:

Band-based similarity indices for gene expression classification and clustering

Aurora Torrente[1]

[1] *Instituto Gregorio Millán, Departamento de Matemáticas*
Universidad Carlos III de Madrid
Av. Universidad 30, 28911 Leganés, Spain
e-mail: etorrent@est-econ.uc3m.es

Scientific Reports

1 Simulated data: analysis of consistency

1.1 Classification

Figure S1: Consistency of the classification error rates for kNN as J increases, in simulated data. All band-based dissimilarity measures, except RR, are very stable with respect to these rates, which are low in general and make them suitable for classification tasks. S and O are consistently the best options.

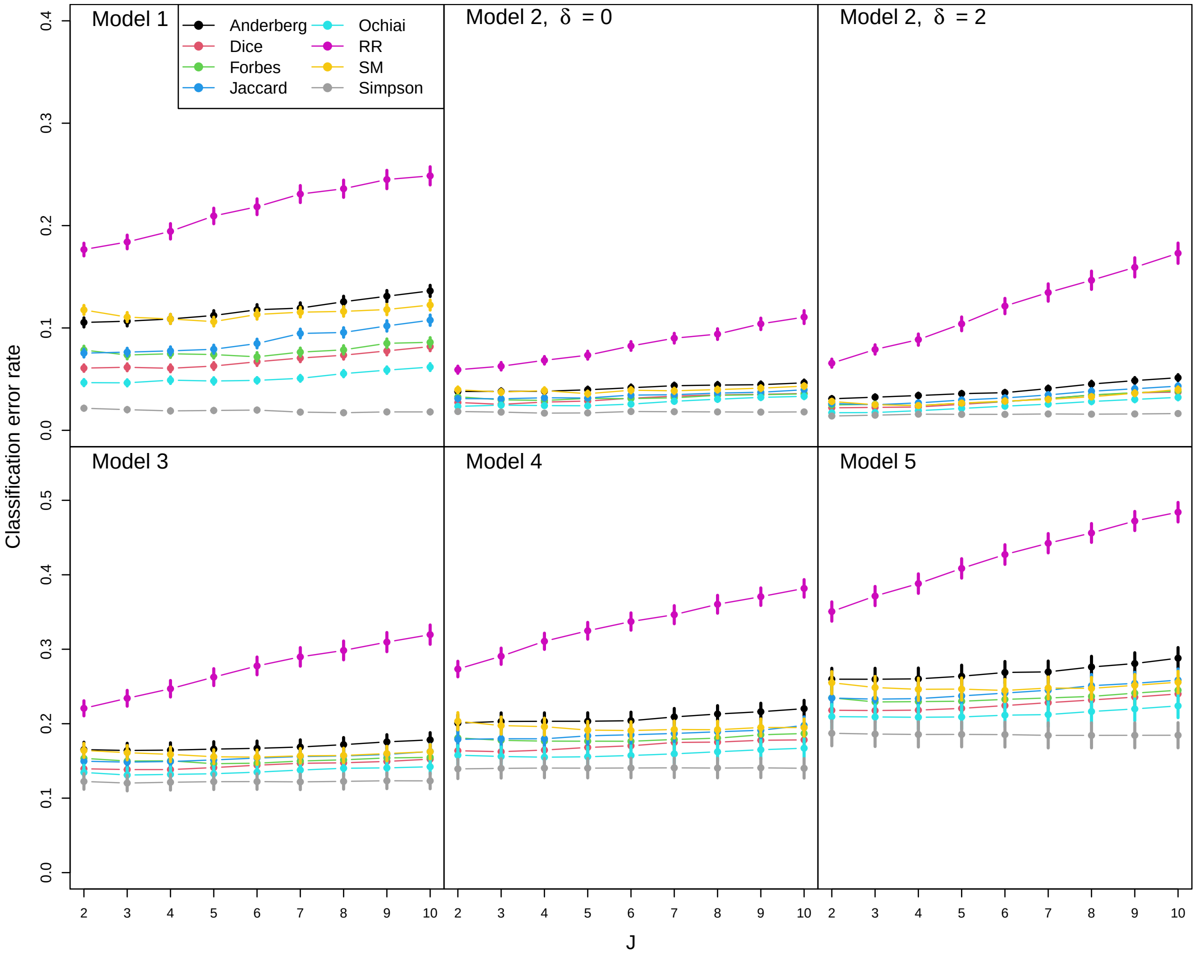

1.2 Clustering

Figure S2: Consistency of clustering error rates produced by the PAM algorithm as J increases, for simulated data. All band-based dissimilarity measures produce stable outputs for all the models, but the performance deteriorates as J increases, specially for RR. The error rates rank the methods from best (S) to worst (RR), regardless of the model. S appears as the only sound technique for this variety of datasets.

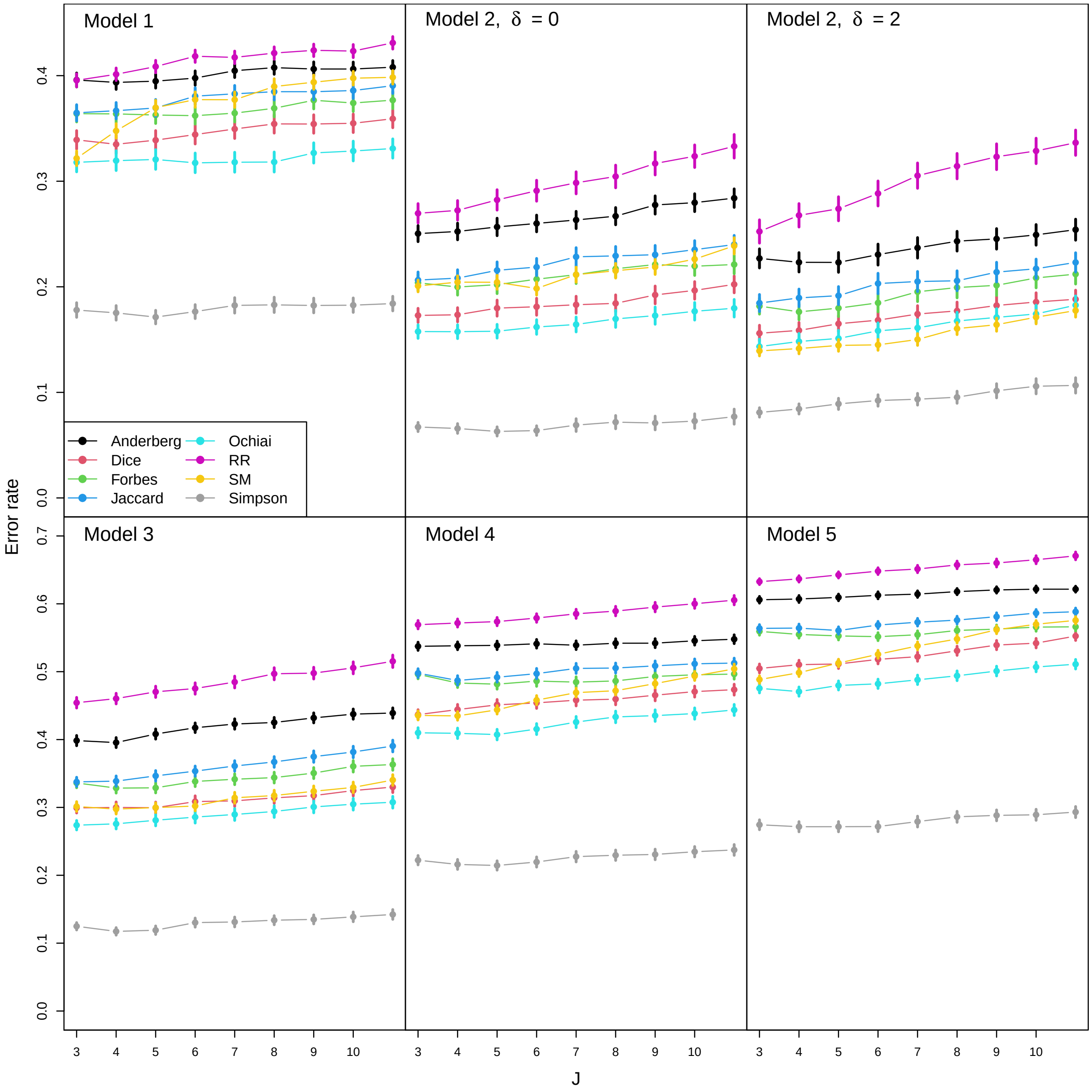

Figure S3: Consistency of ARI values produced by the PAM algorithm as J increases, for simulated data. All the band-based dissimilarity measures lead to stable results, but there is a trend to deteriorate the performance for larger values of J, whose strength depends on the particular method. Likewise to the error rate case, ARI allows ranking the methods from best (S) to worst (RR) for all datasets.

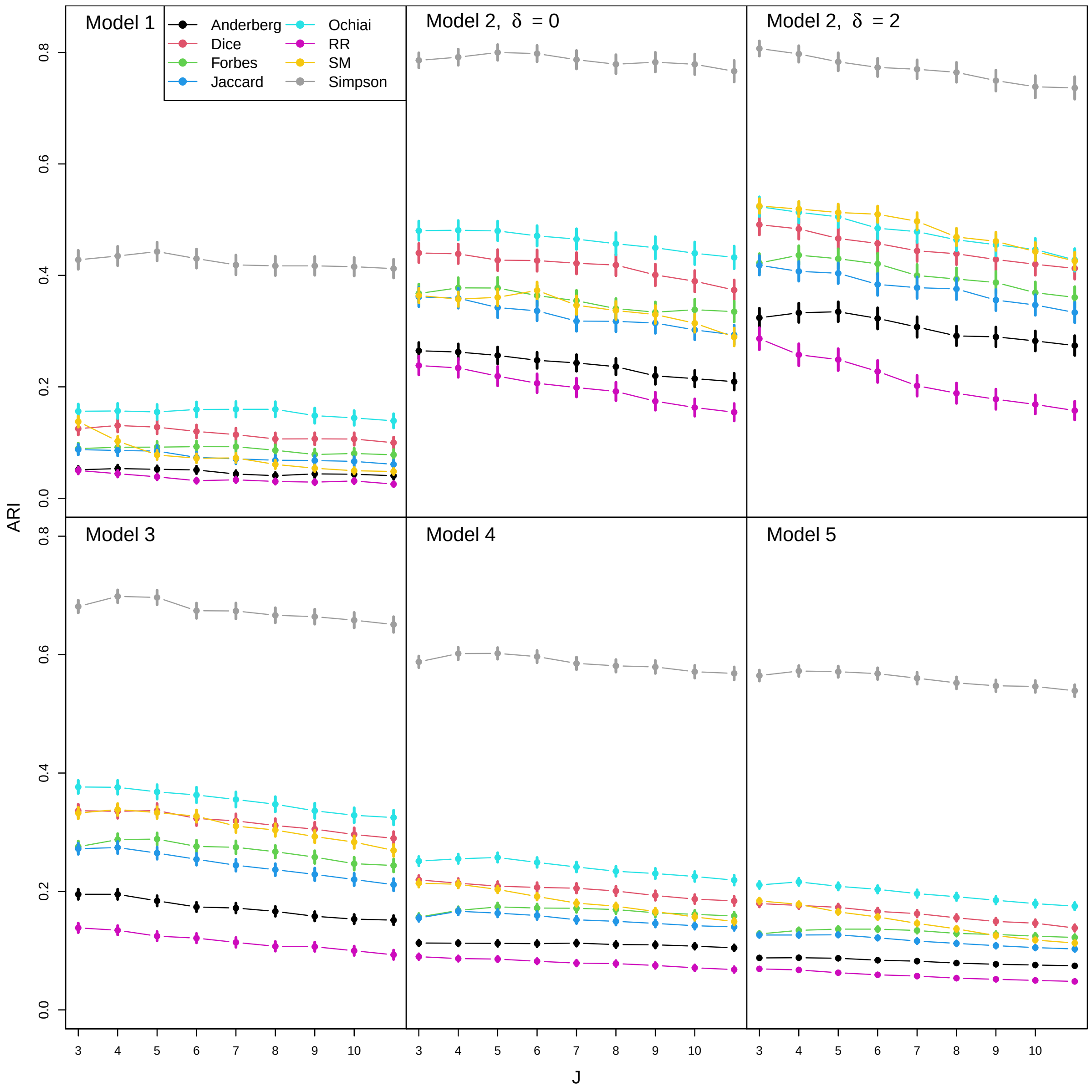

2 Real data

2.1 Lymphoma dataset

Figure S4: Clustering the lymphoma data set. Dendrograms and heatmaps for the lymphoma data set, using the band-based indices (for $J = 2$ and 3) and the classical distances. The colour labels indicate the sample types.

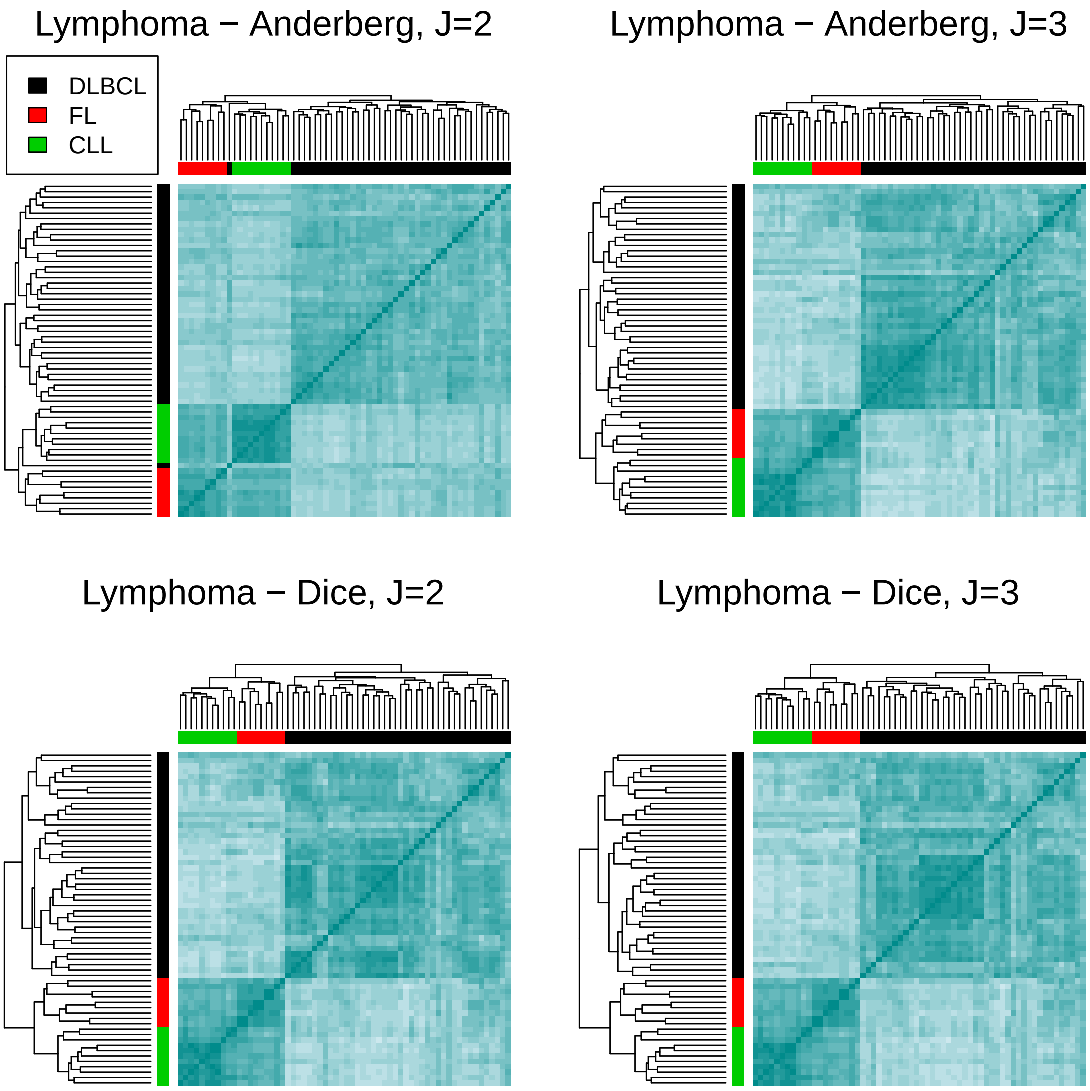

Lymphoma − Forbes, J=2

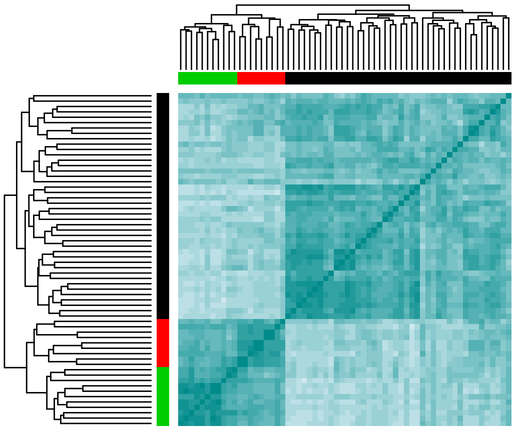

Lymphoma − Forbes, J=3

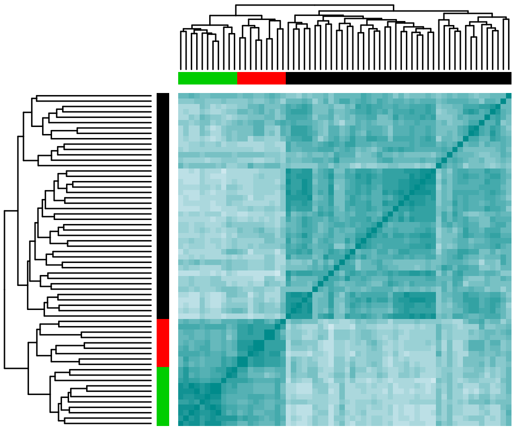

Lymphoma − Jaccard, J=2

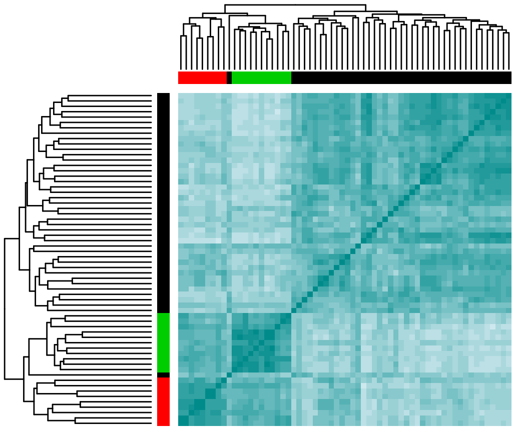

Lymphoma − Jaccard, J=3

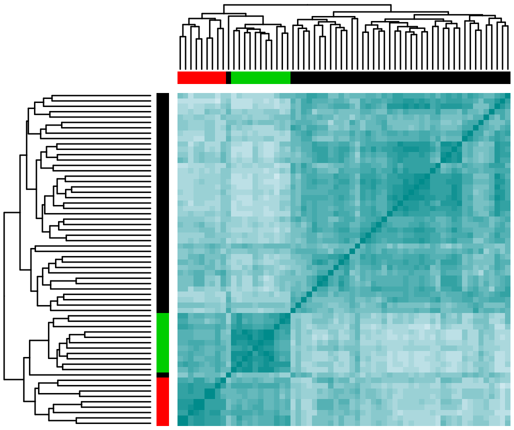

Lymphoma − Simpson, J=3

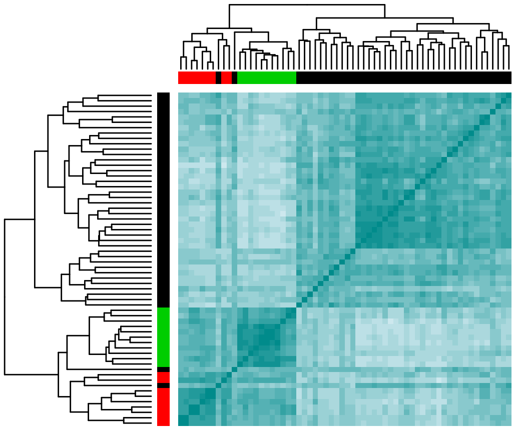

Lymphoma − Ochiai, J=3

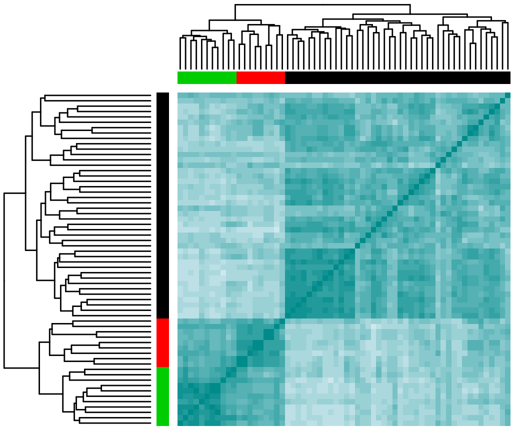

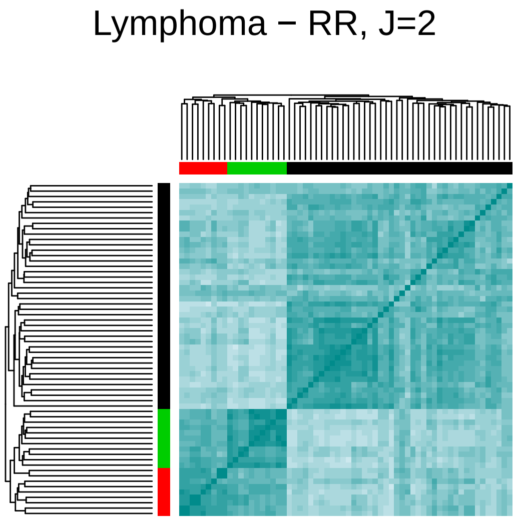
Lymphoma − RR, J=2

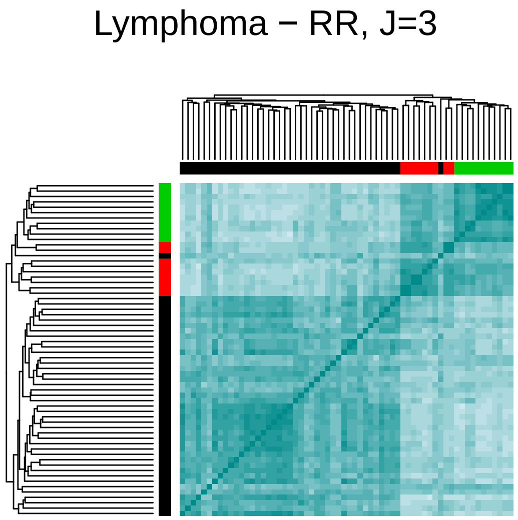
Lymphoma − RR, J=3

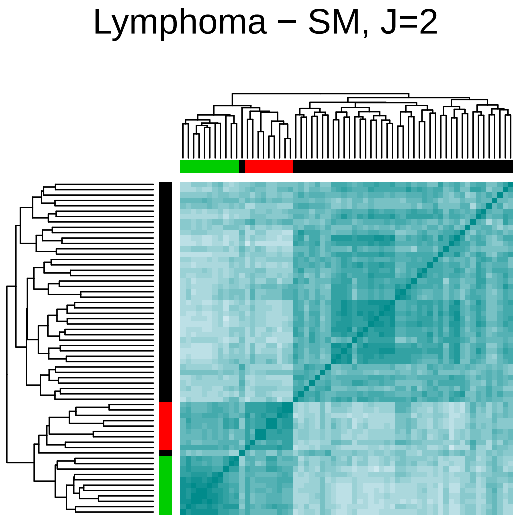
Lymphoma − SM, J=2

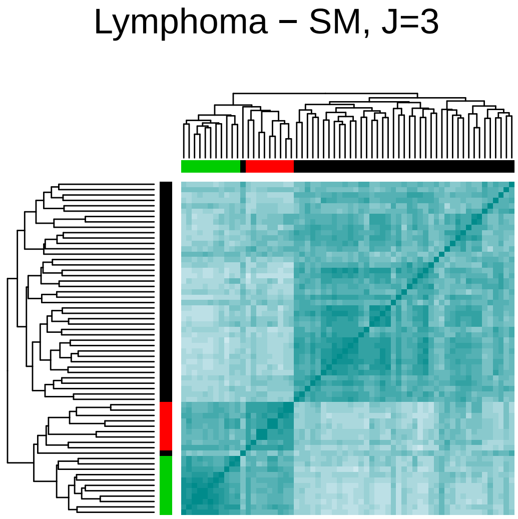
Lymphoma − SM, J=3

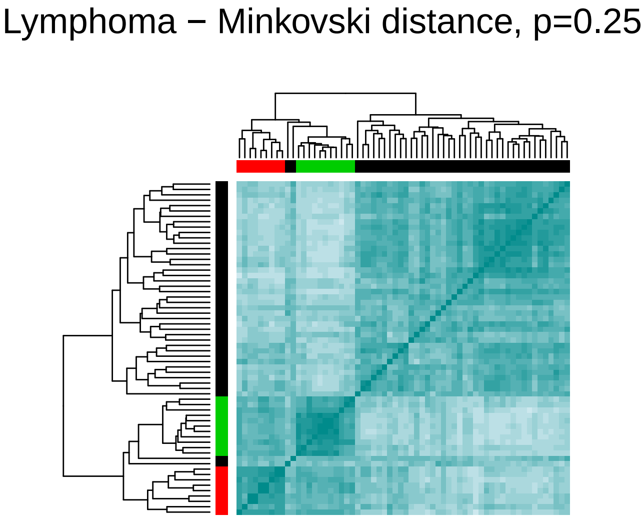
Lymphoma − Minkovski distance, p=0.25

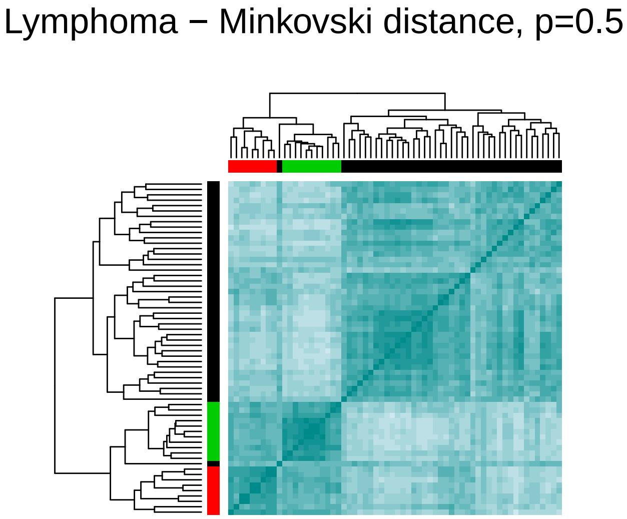
Lymphoma − Minkovski distance, p=0.5

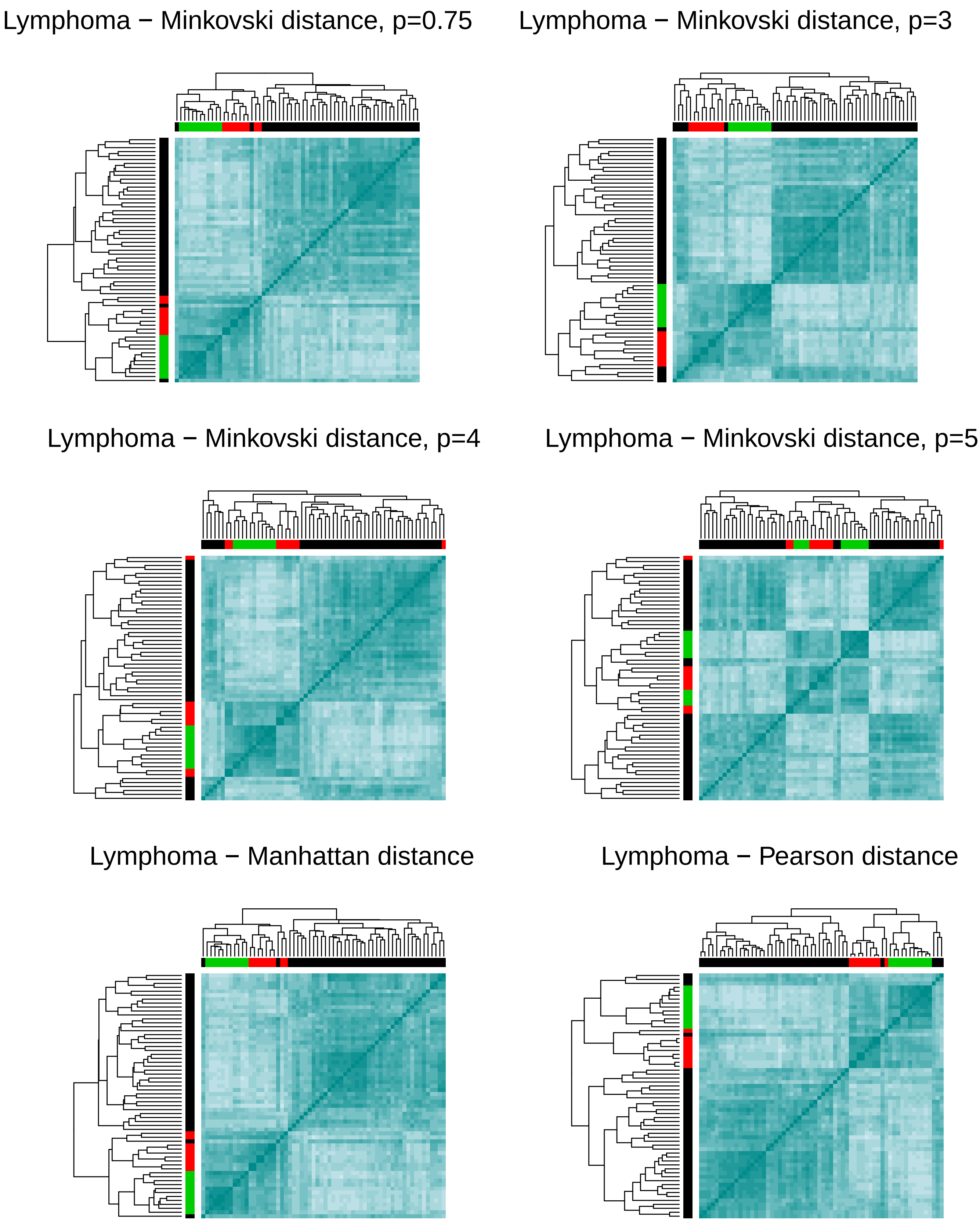
Lymphoma − Minkovski distance, p=0.75
Lymphoma − Minkovski distance, p=3
Lymphoma − Minkovski distance, p=4
Lymphoma − Minkovski distance, p=5
Lymphoma − Manhattan distance
Lymphoma − Pearson distance

2.2 Colon dataset

Figure S5: Clustering the colon data set. Dendrograms and heatmaps for the colon data set, using the band-based indices (for $J = 2$ and 3) and the classical distances. The colour labels indicate the sample types.

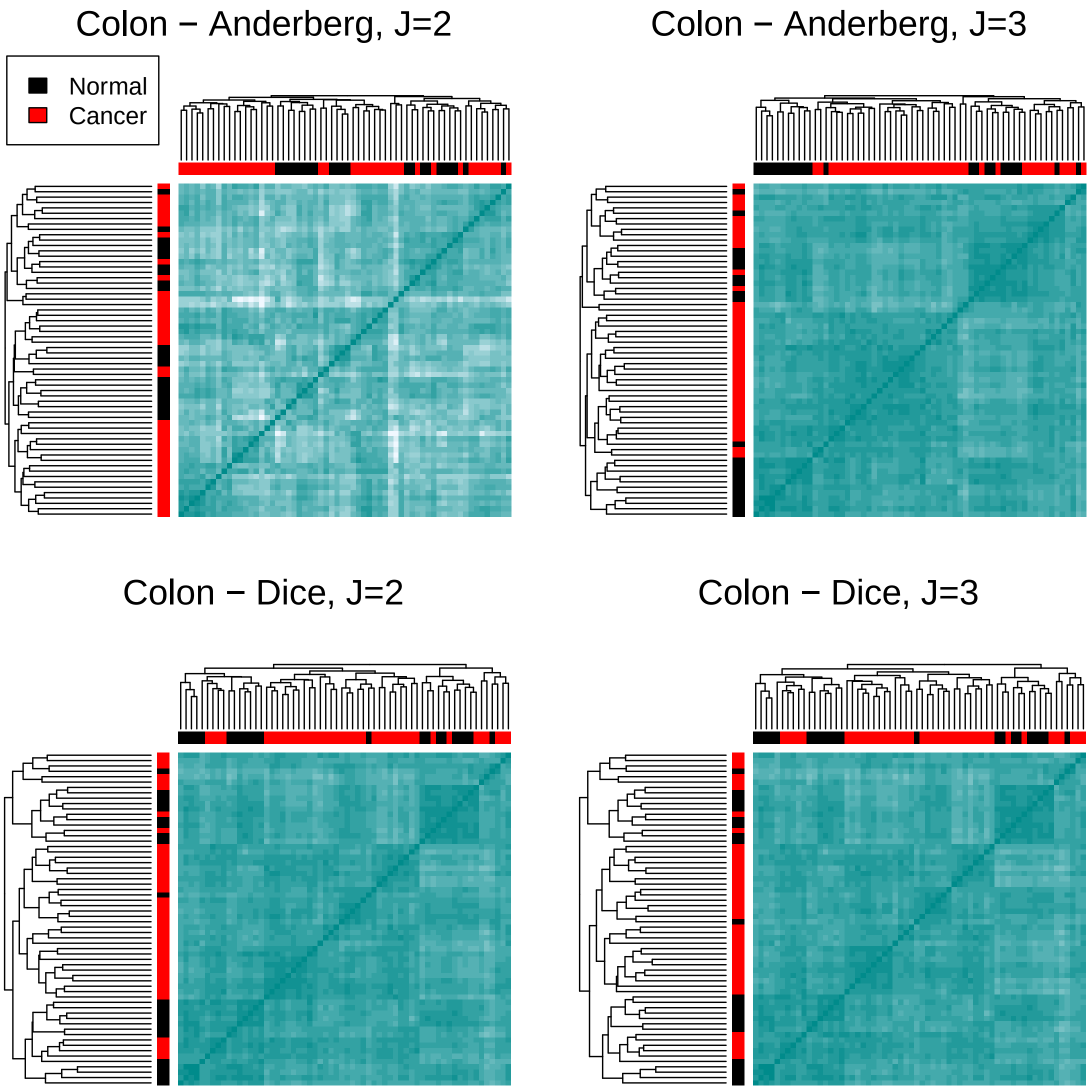

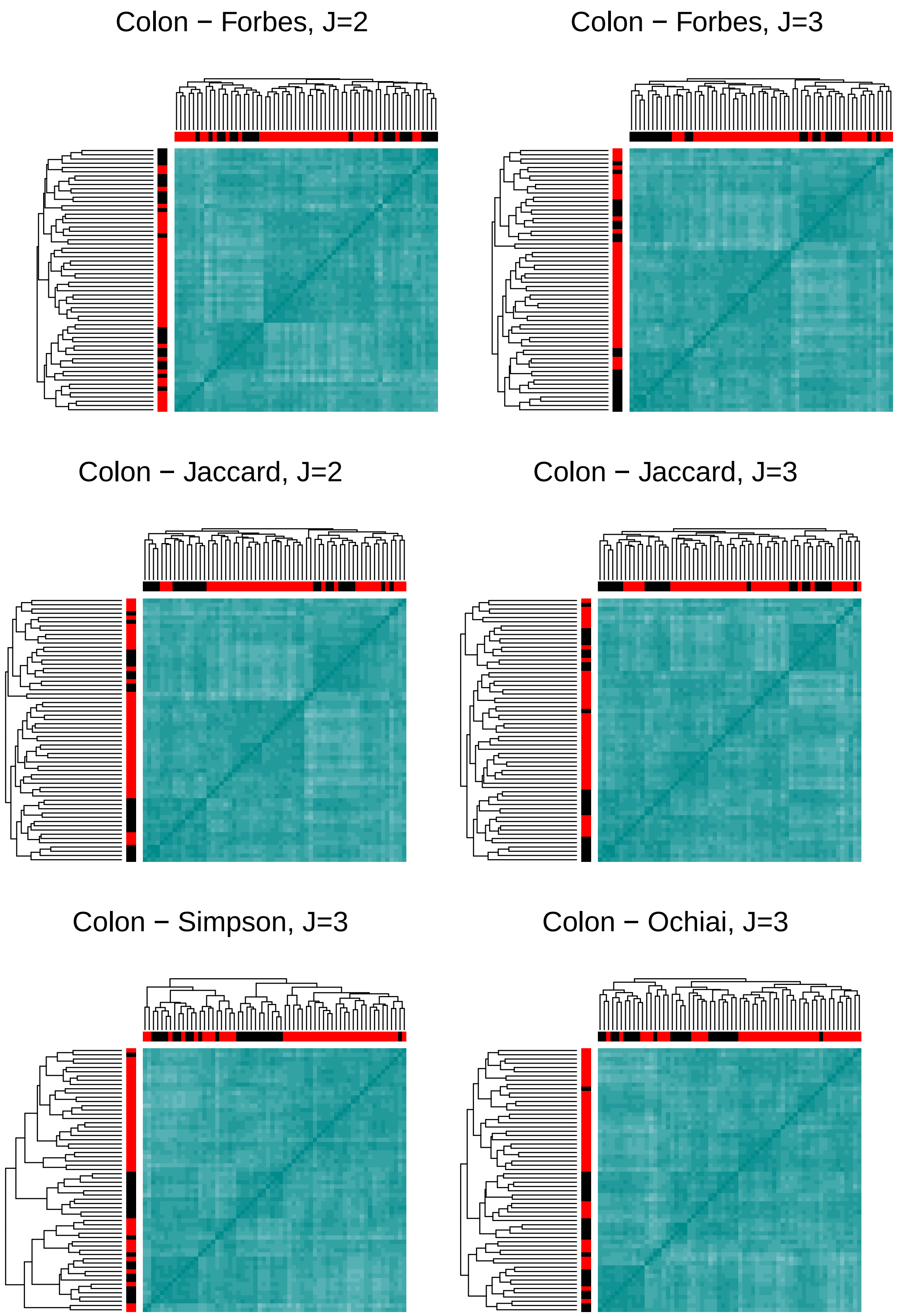
Colon − Forbes, J=2
Colon − Forbes, J=3
Colon − Jaccard, J=2
Colon − Jaccard, J=3
Colon − Simpson, J=3
Colon − Ochiai, J=3

Colon − RR, J=2

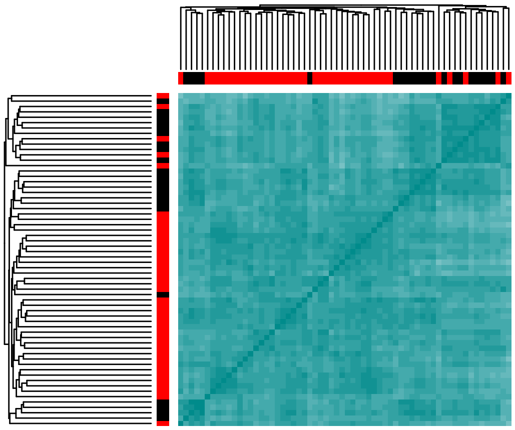

Colon − RR, J=3

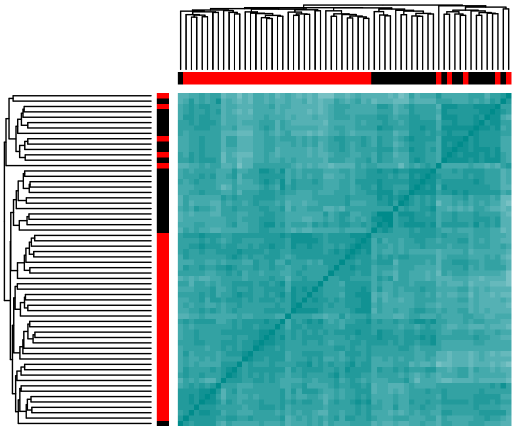

Colon − SM, J=2

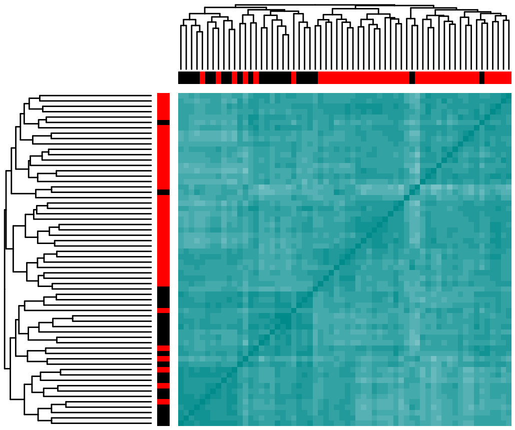

Colon − SM, J=3

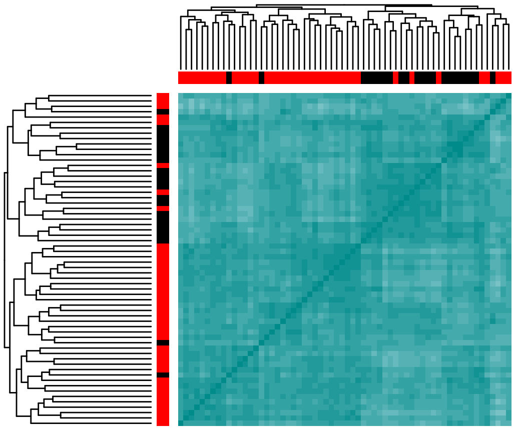

Colon − Minkovski distance, p=0.25

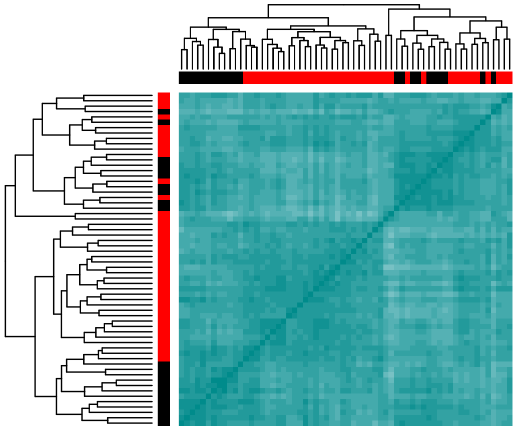

Colon − Minkovski distance, p=0.5

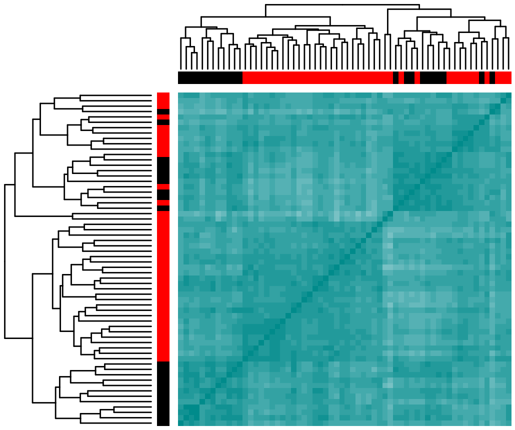

Colon − Minkovski distance, p=0.75

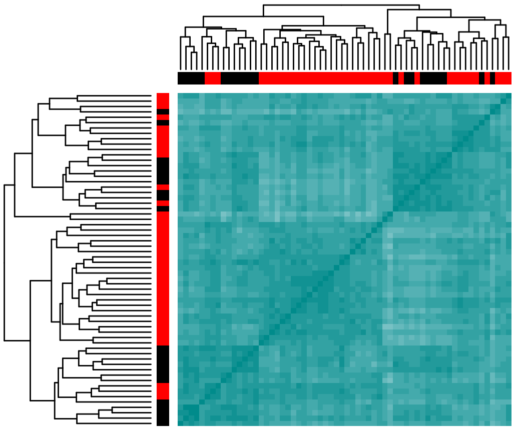

Colon − Minkovski distance, p=3

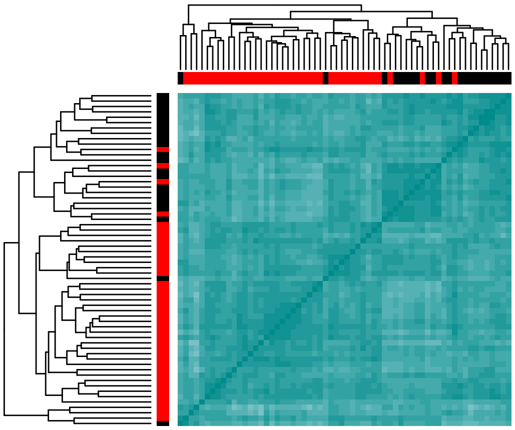

Colon − Minkovski distance, p=4

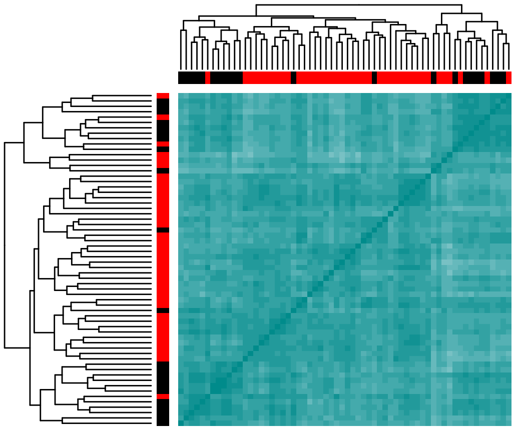

Colon − Minkovski distance, p=5

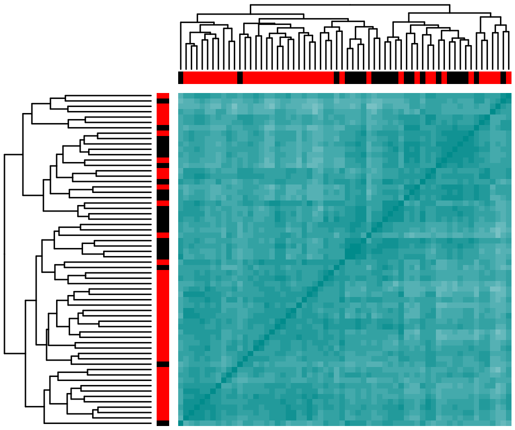

Colon − Manhattan distance

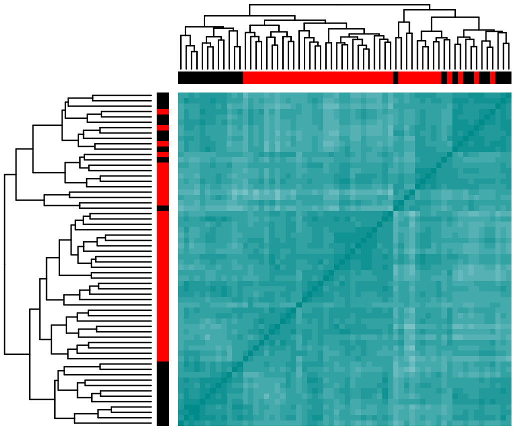

Colon − Pearson distance

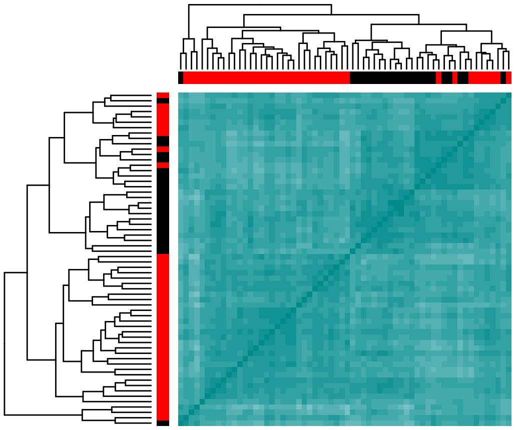

2.3 Leukemia dataset

Figure S6: Clustering the leukemia data set. Dendrograms and heatmaps for the leukemia data set, using the band-based indices (for $J = 2$ and 3) and the classical distances. The colour labels indicate the sample types according to the 2-class or 3-class cases.

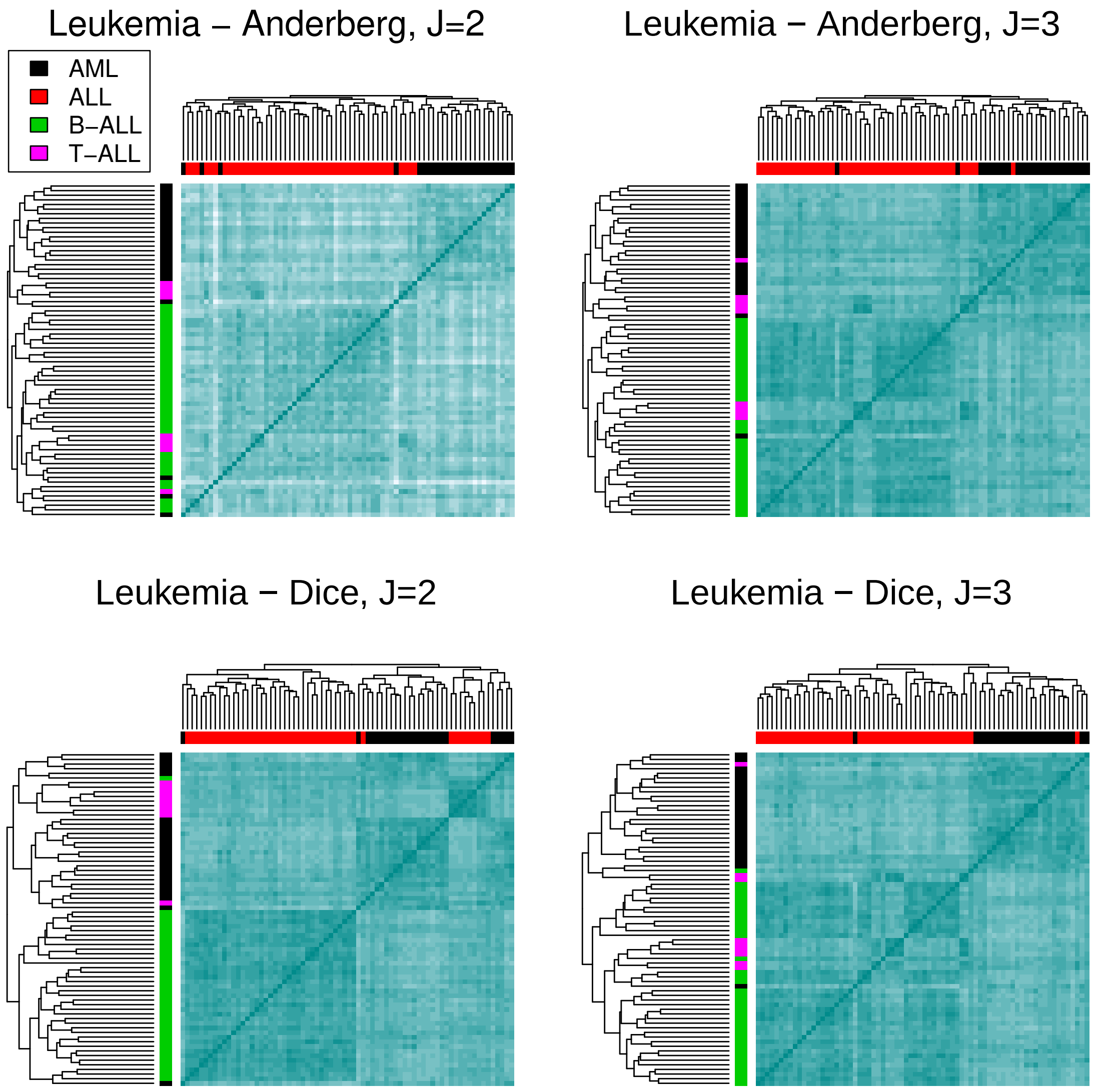

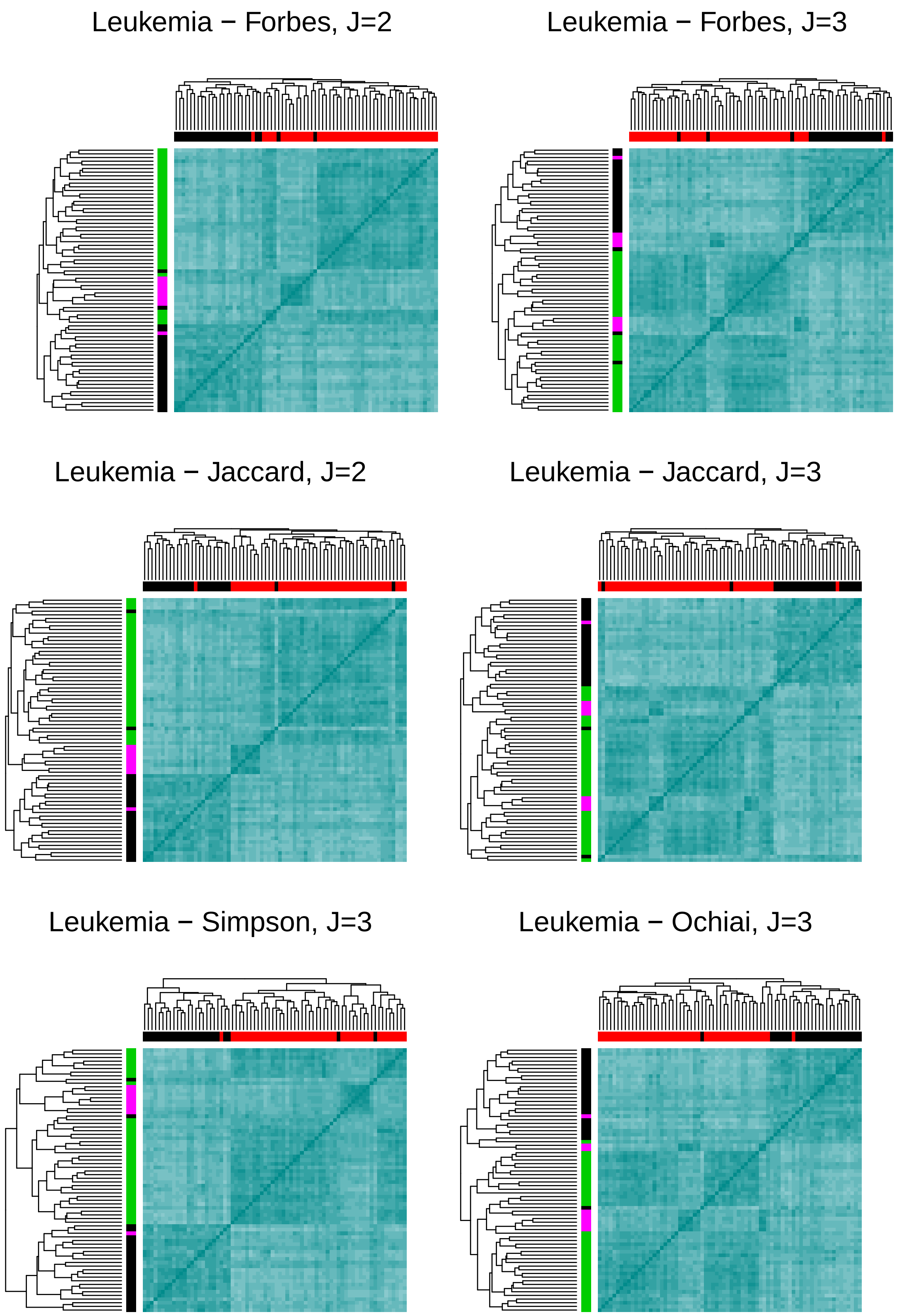
Leukemia − Forbes, J=2
Leukemia − Forbes, J=3
Leukemia − Jaccard, J=2
Leukemia − Jaccard, J=3
Leukemia − Simpson, J=3
Leukemia − Ochiai, J=3

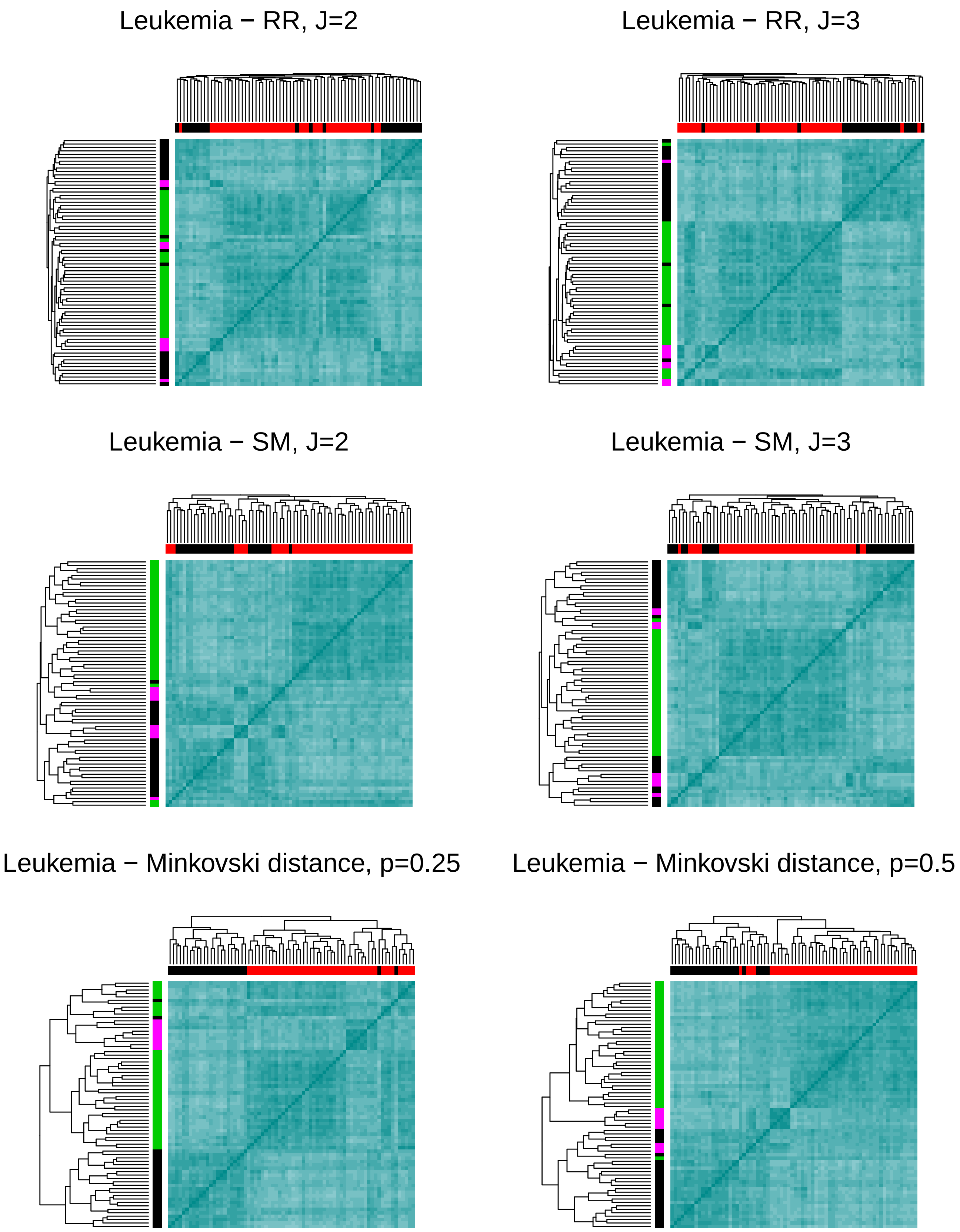
Leukemia − RR, J=2
Leukemia − RR, J=3
Leukemia − SM, J=2
Leukemia − SM, J=3
Leukemia − Minkovski distance, p=0.25
Leukemia − Minkovski distance, p=0.5

Leukemia − Minkovski distance, p=0.75

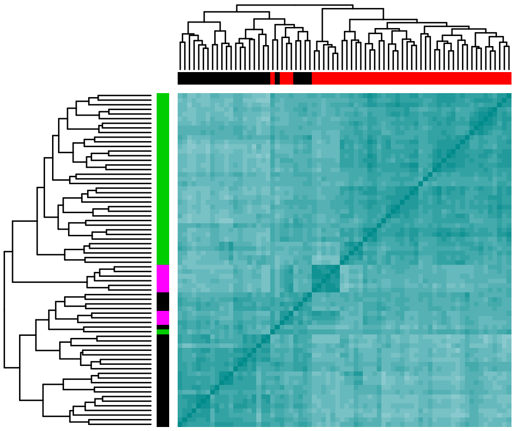

Leukemia − Minkovski distance, p=3

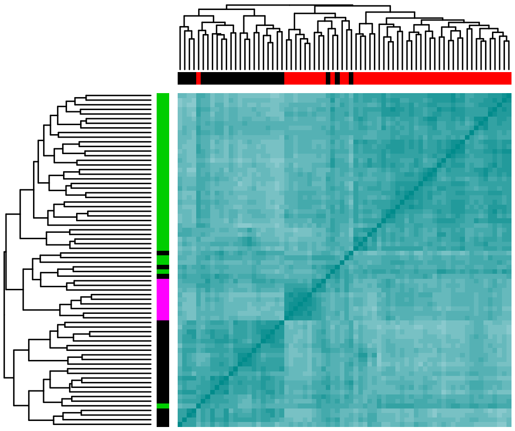

Leukemia − Minkovski distance, p=4

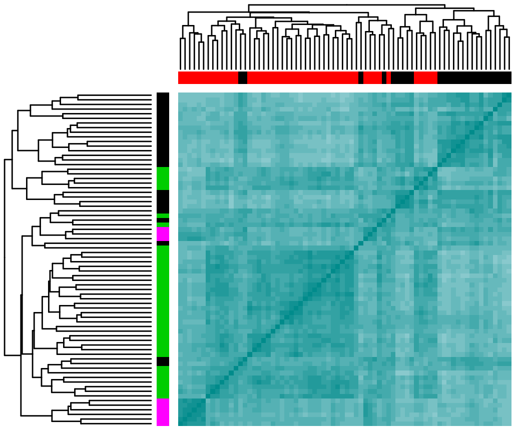

Leukemia − Minkovski distance, p=5

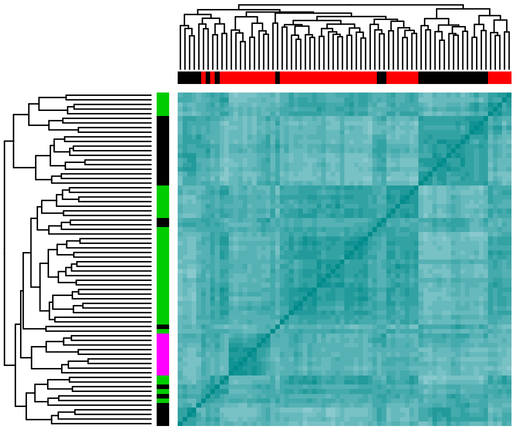

Leukemia − Manhattan distance

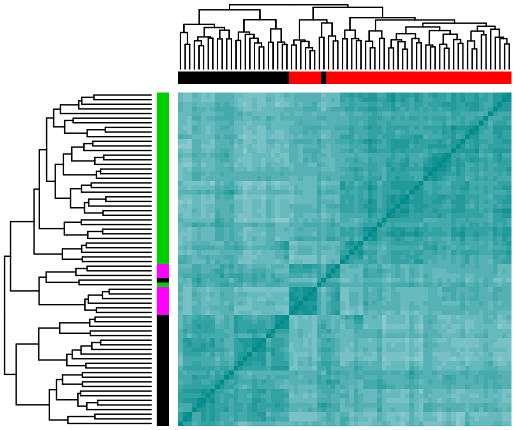

Leukemia − Pearson distance

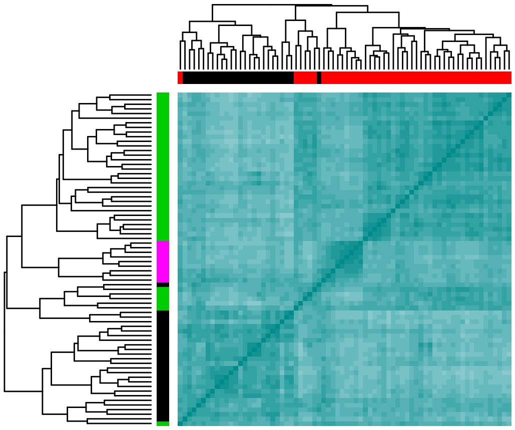

2.4 Pan-cancer dataset

Figure S7: Clustering the pan-cancer data set. Dendrograms for the pan-cancer data set, using the band-based indices (for $J = 2$ and 3) and the classical distances. The colour labels indicate the cancer types.

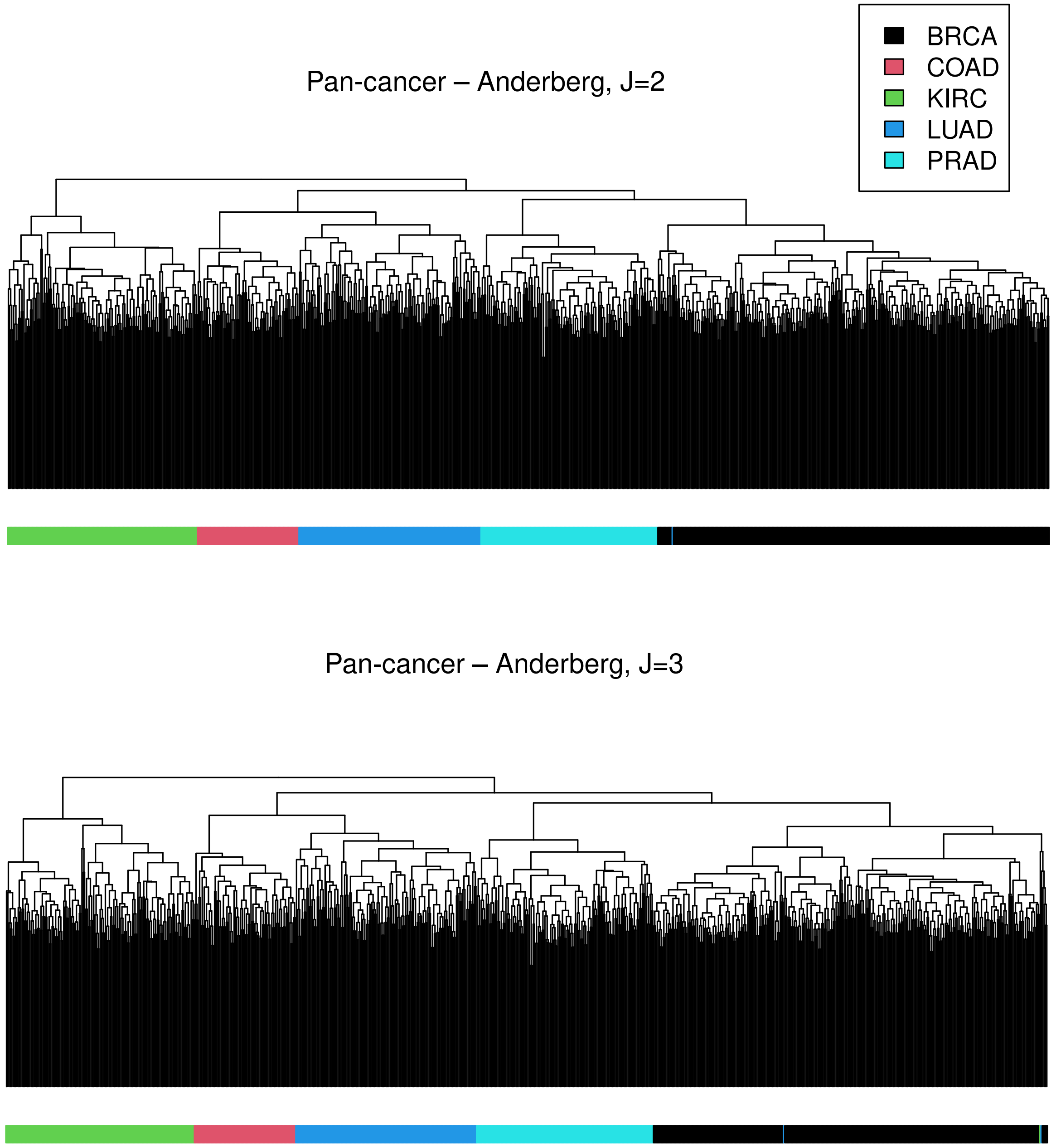

Pan-cancer – Dice, J=2

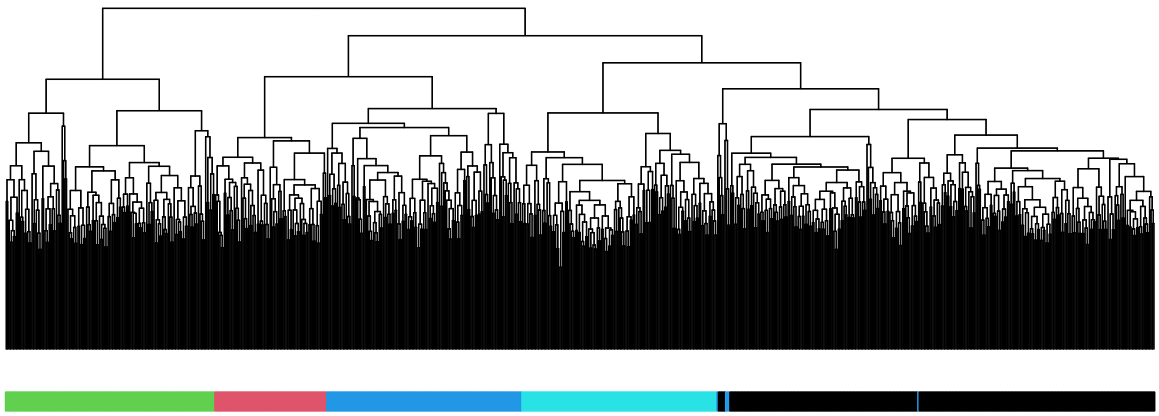

Pan-cancer – Dice, J=3

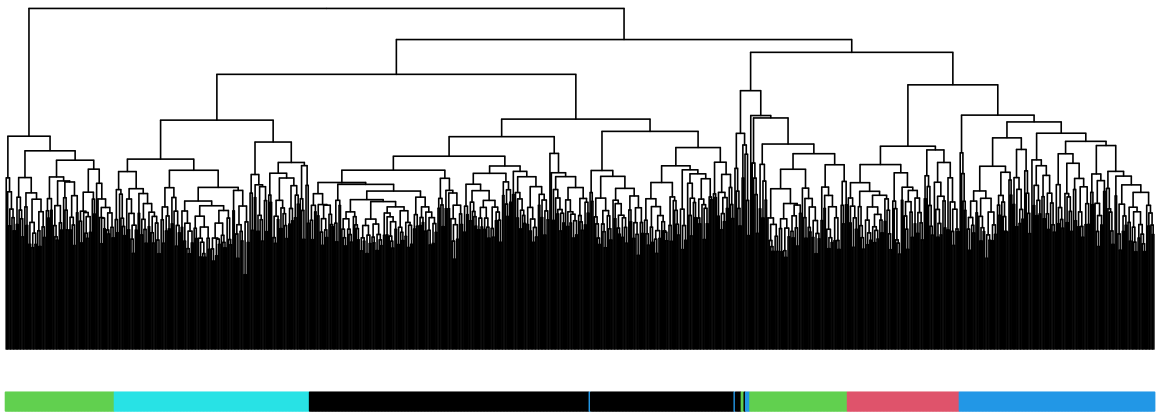

Pan-cancer – Forbes, J=2

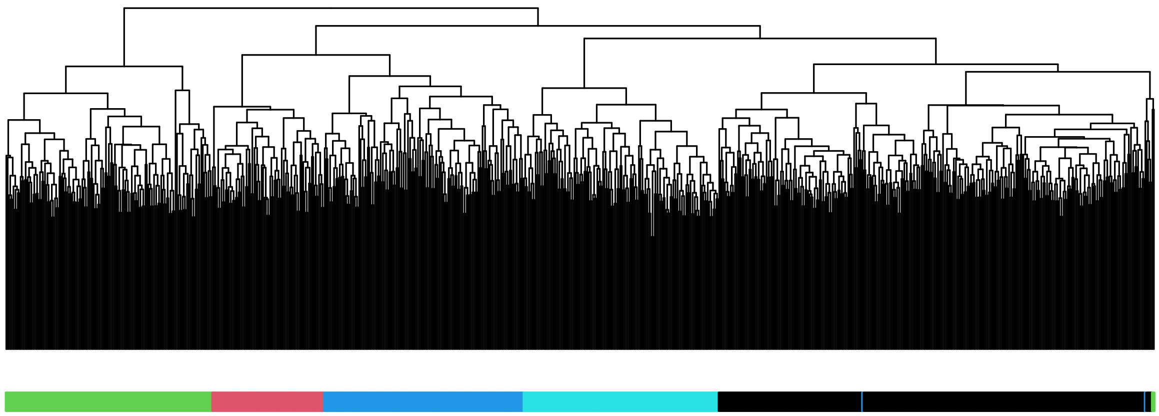

Pan-cancer – Forbes, J=3

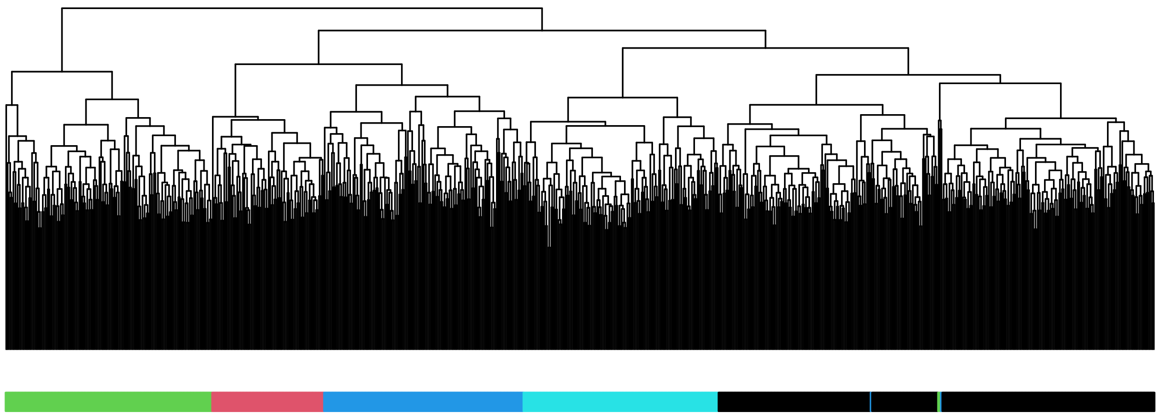

Pan-cancer – Jaccard, J=2

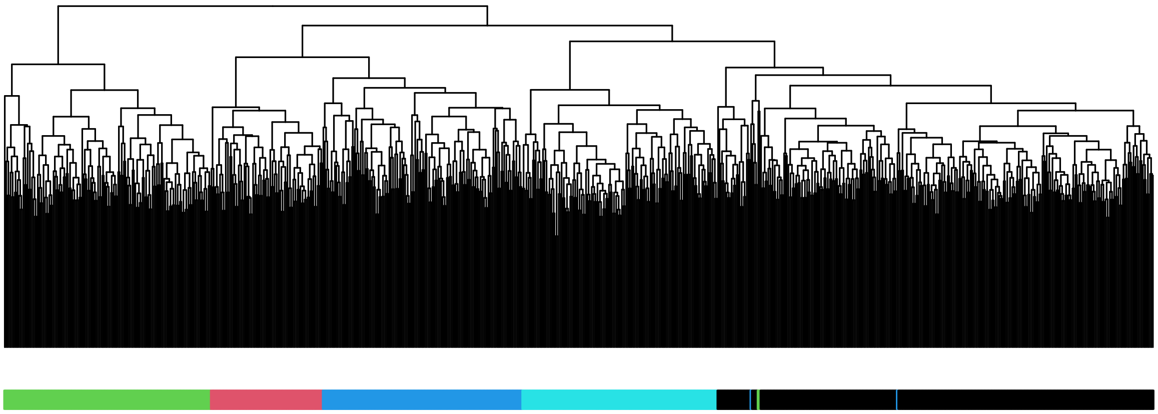

Pan-cancer – Jaccard, J=3

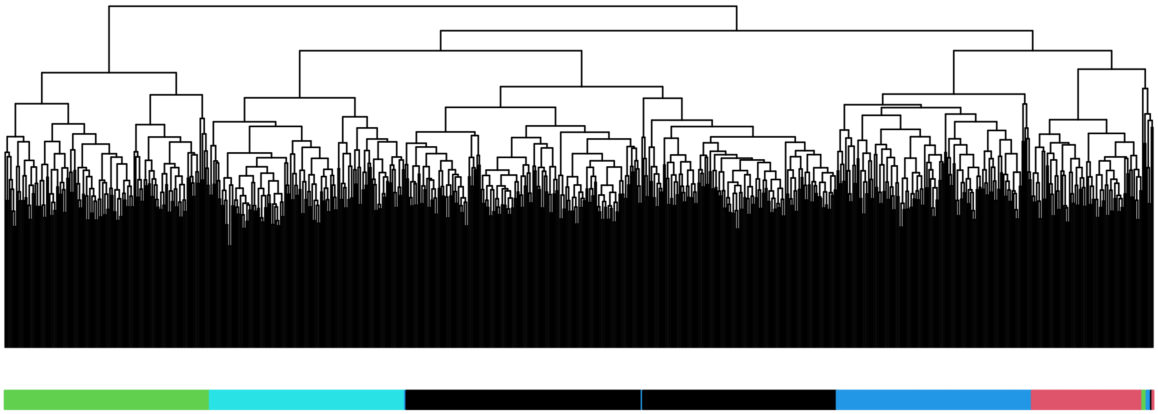

Pan-cancer – Simpson, J=3

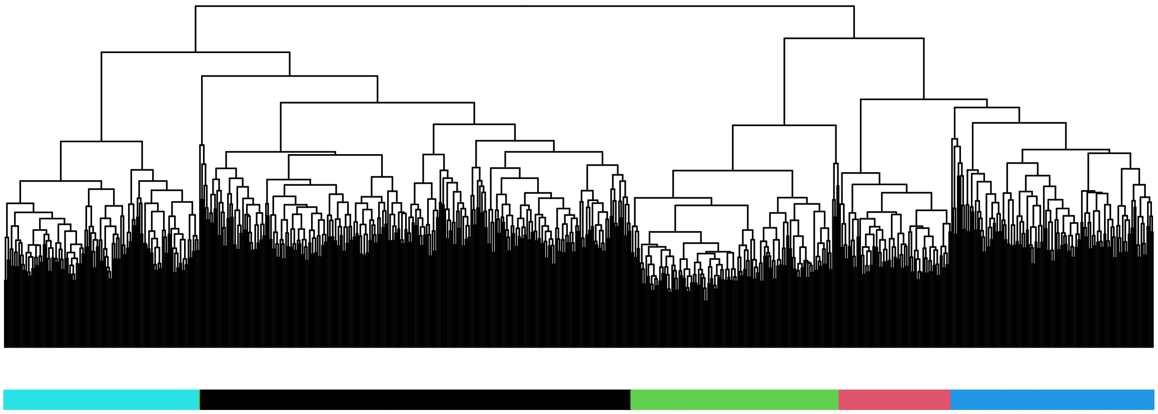

Pan-cancer – Ochiai, J=3

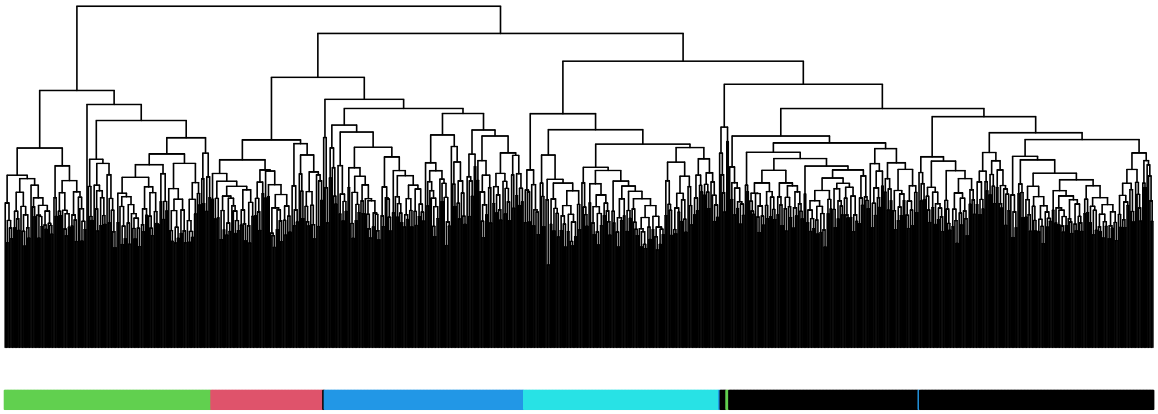

Pan-cancer – RR, J=2

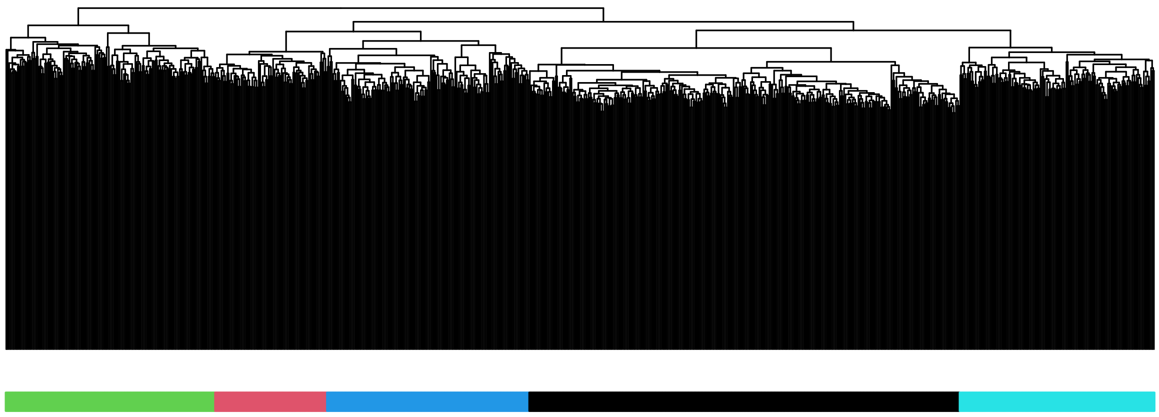

Pan-cancer – RR, J=3

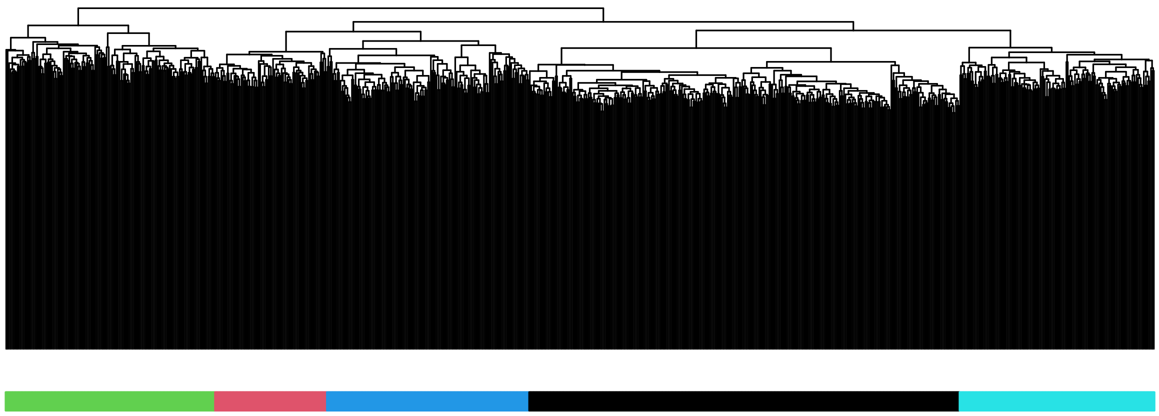

Pan-cancer – SM, J=2

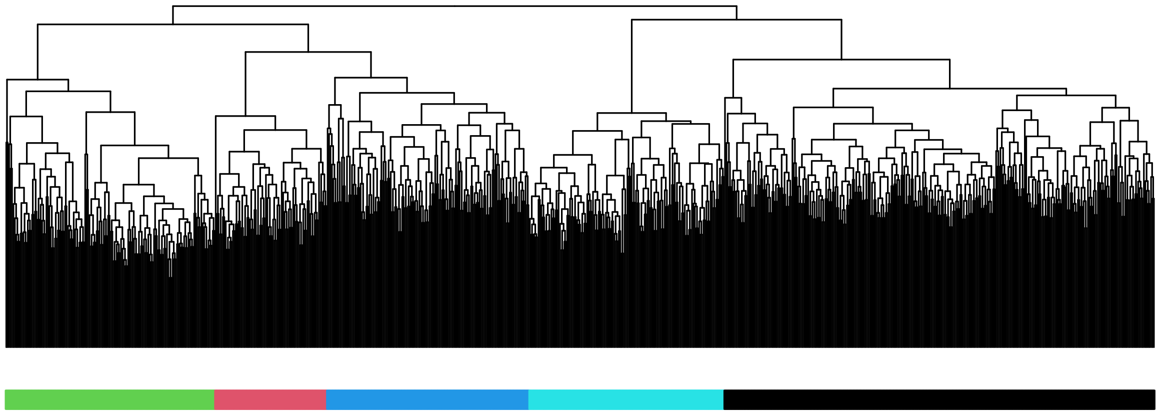

Pan-cancer – SM, J=3

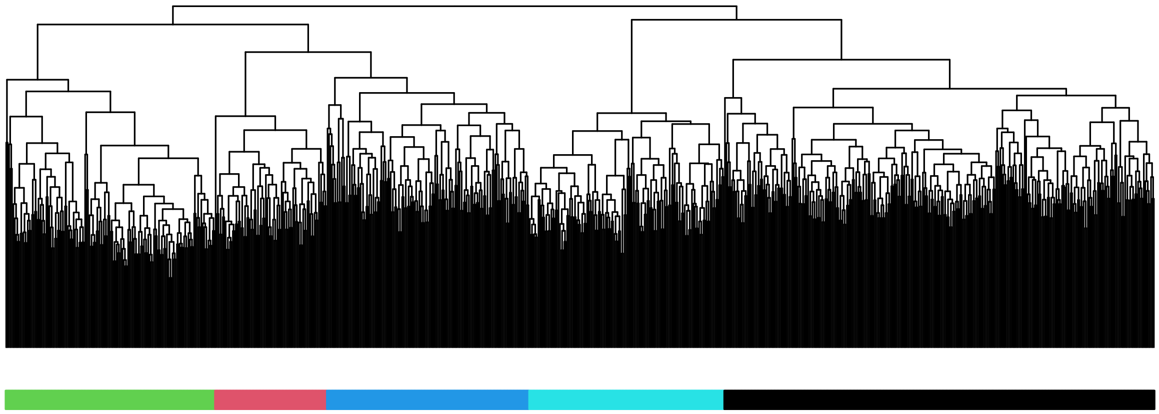

Pan-cancer – Minkovski distance, p=0.25

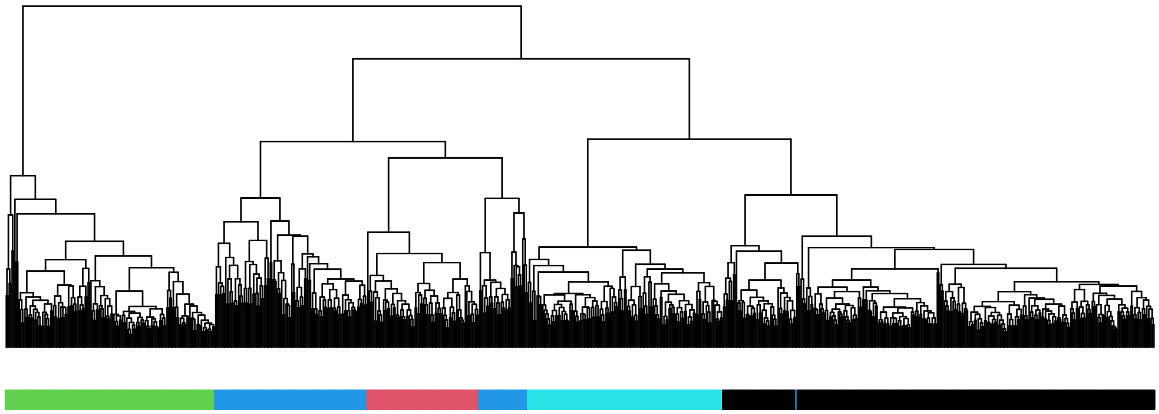

Pan-cancer – Minkovski distance, p=0.5

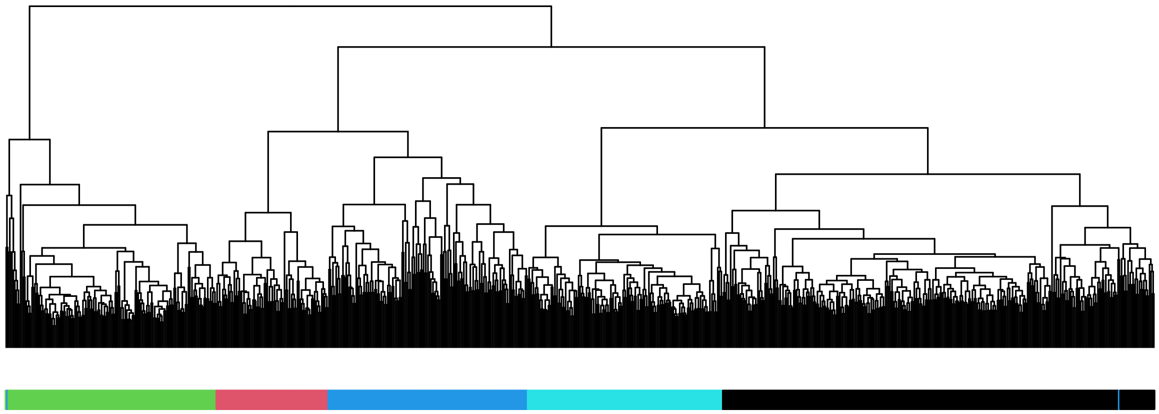

Pan-cancer – Minkovski distance, p=0.75

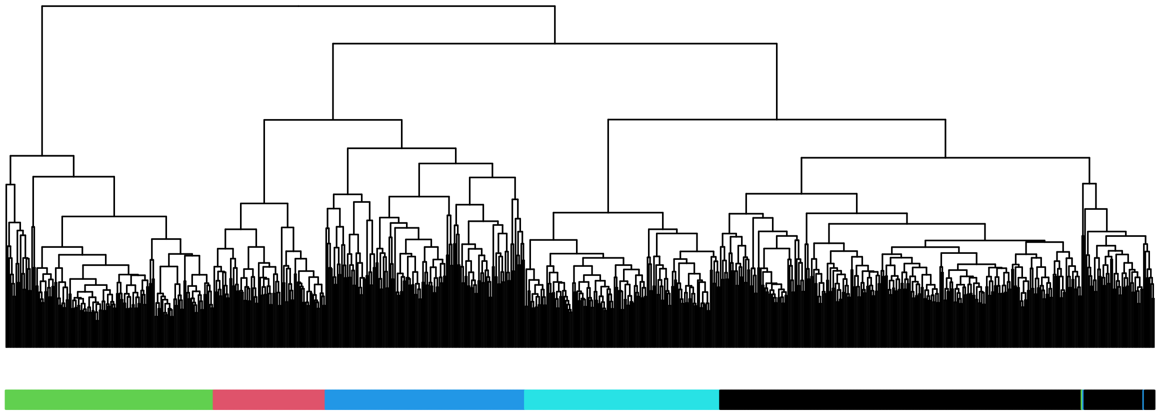

Pan-cancer – Minkovski distance, p=3

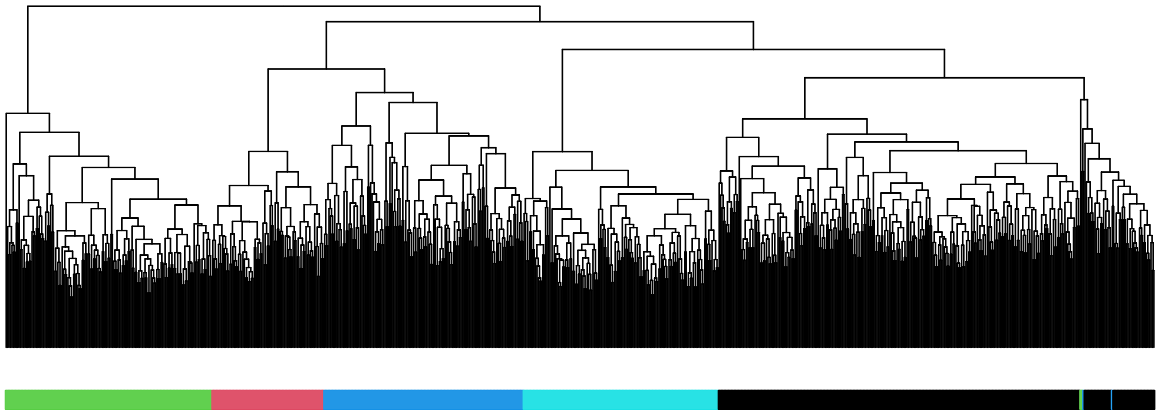

Pan-cancer – Minkovski distance, p=4

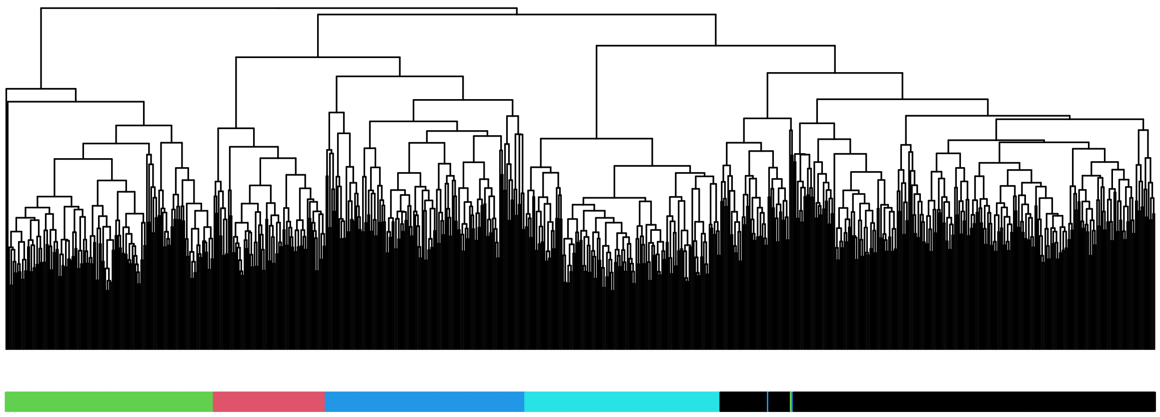

Pan-cancer – Minkovski distance, p=5

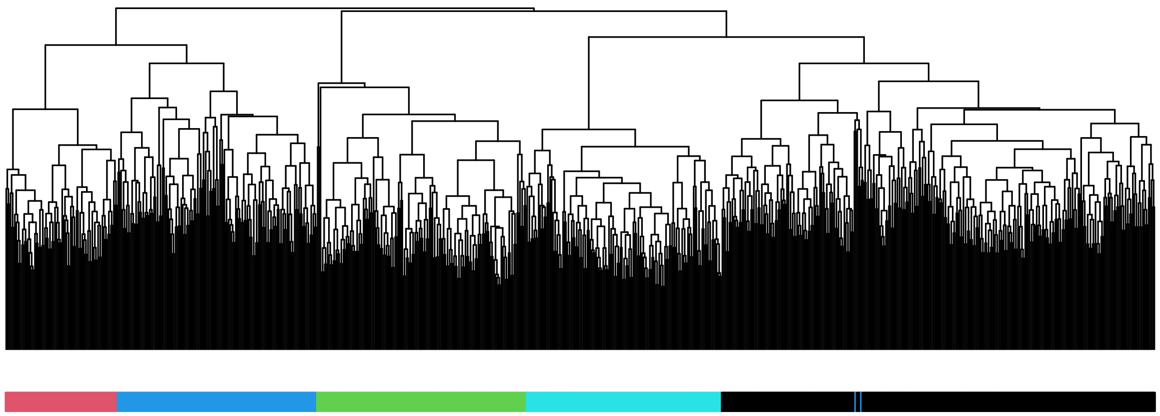

Pan-cancer – Manhattan distance

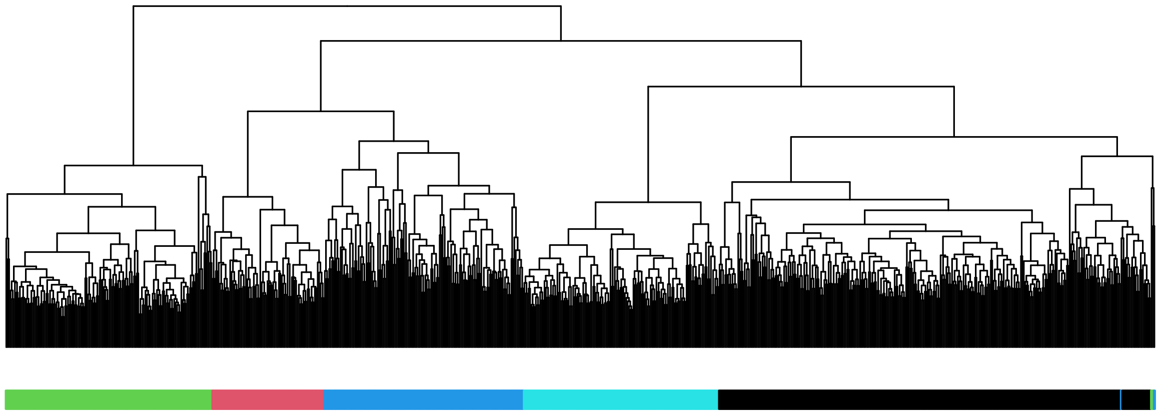

Pan-cancer – Pearson distance

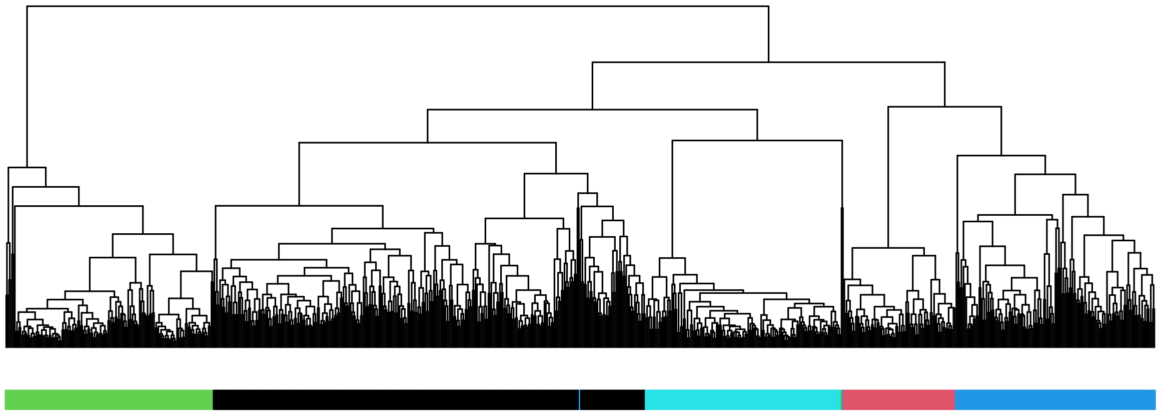

2.5 FFvsFFPE dataset

Figure S8: Clustering the FFvsFFPE (storage condition) data set. Dendrograms and heatmaps for the FFvsFFPE data set, using the band-based indices (for $J = 2$ and 3) and the classical distances. The colour labels indicate the cancer/storage types.

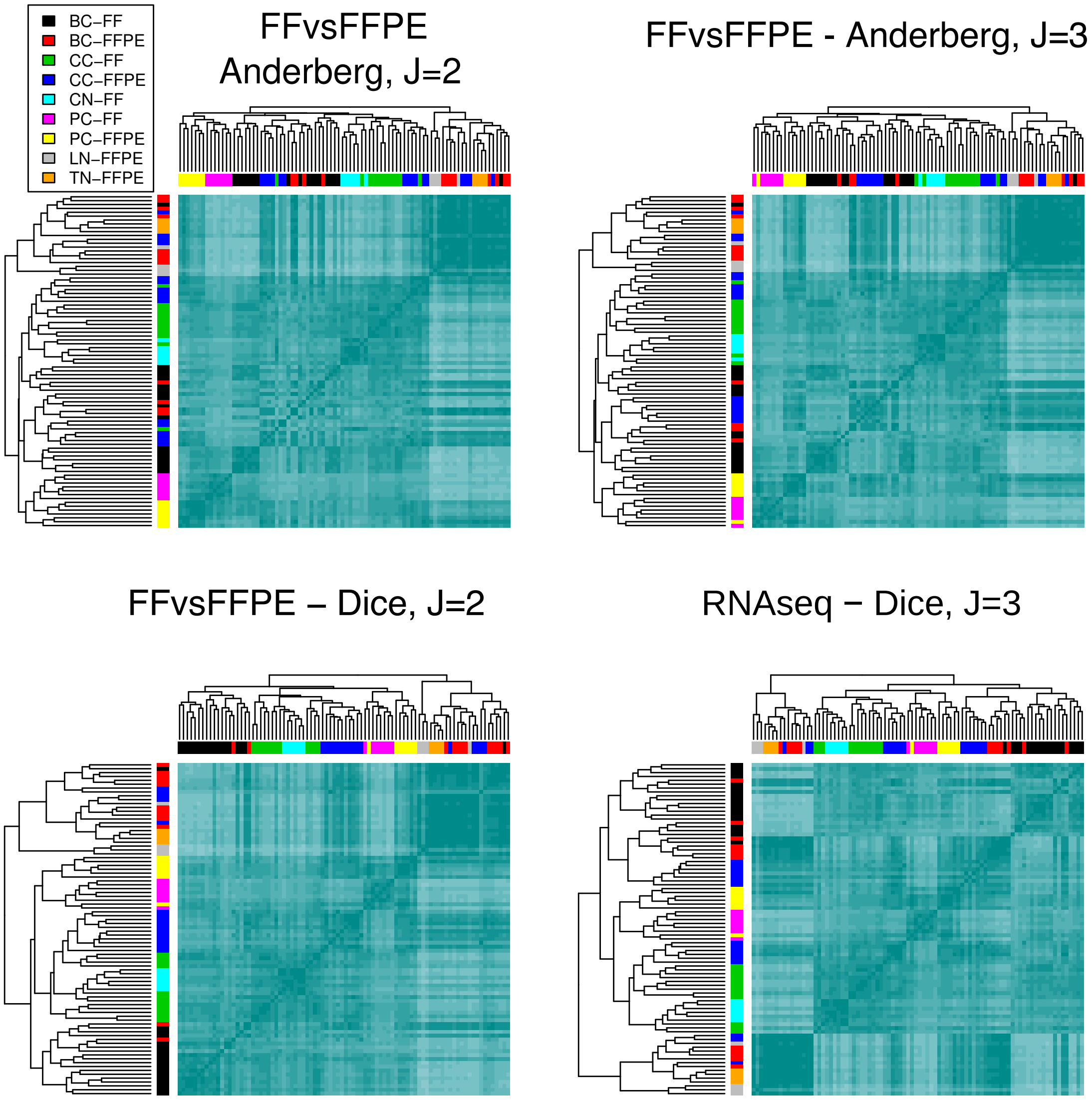

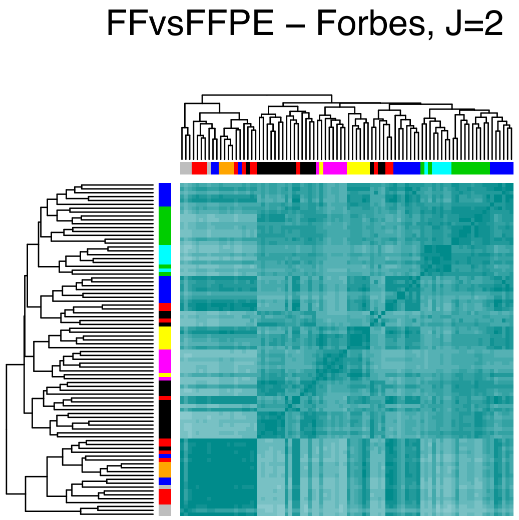
FFvsFFPE – Forbes, J=2

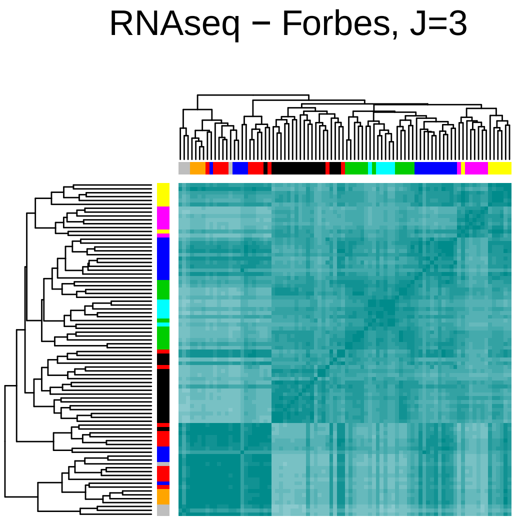
RNAseq − Forbes, J=3

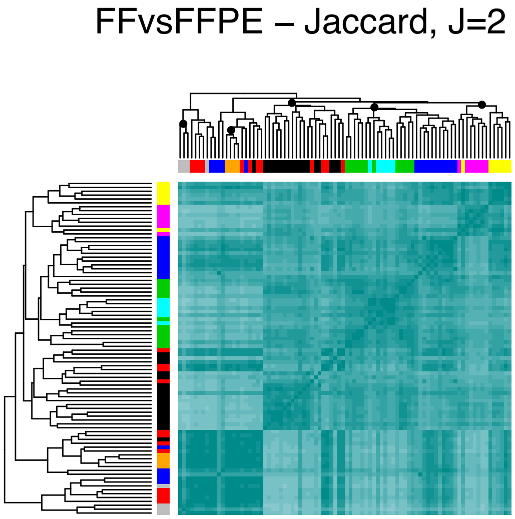
FFvsFFPE – Jaccard, J=2

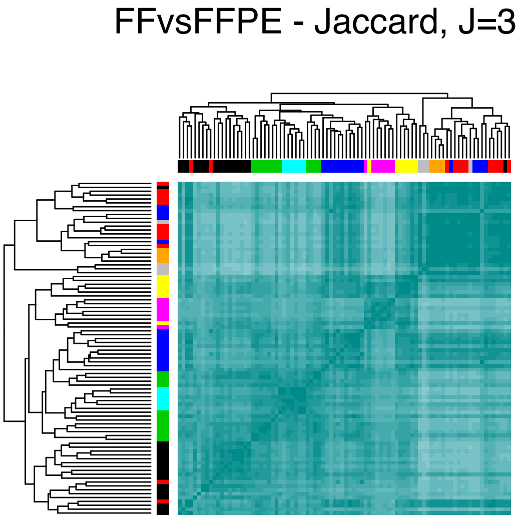
FFvsFFPE - Jaccard, J=3

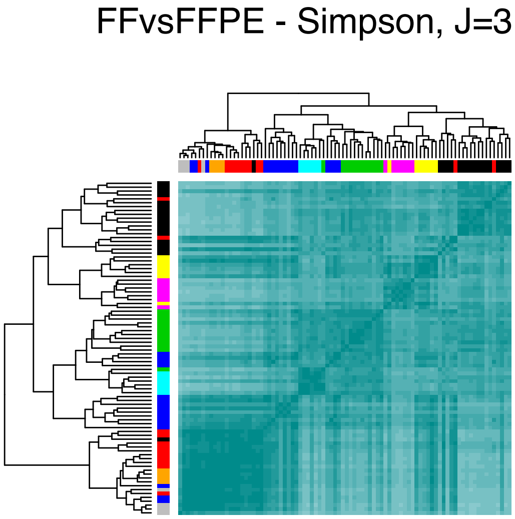
FFvsFFPE - Simpson, J=3

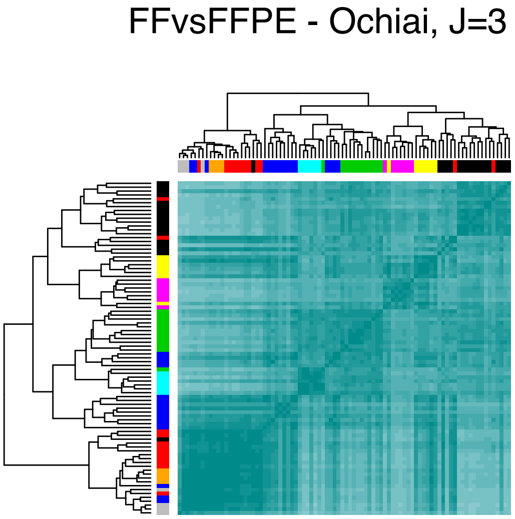
FFvsFFPE - Ochiai, J=3

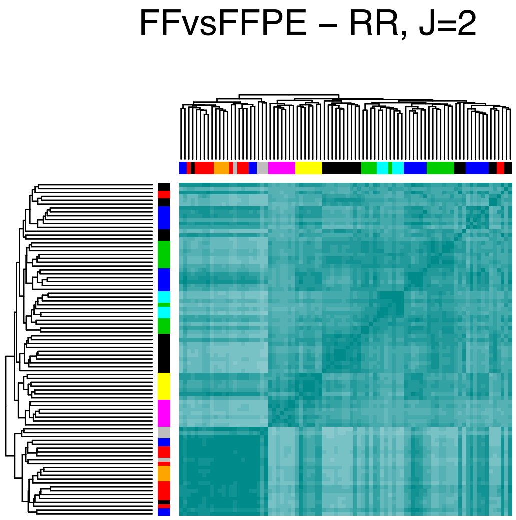
FFvsFFPE – RR, J=2

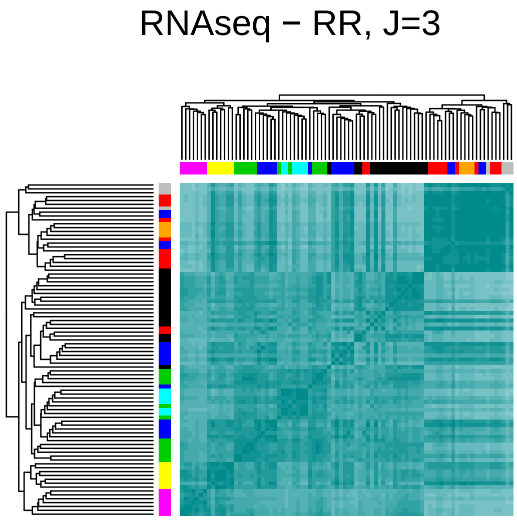
RNAseq − RR, J=3

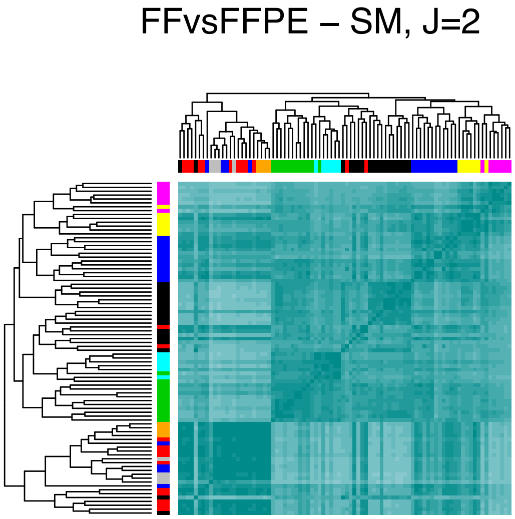
FFvsFFPE – SM, J=2

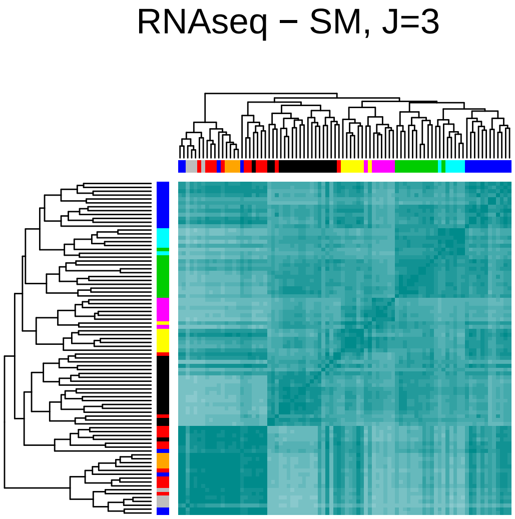
RNAseq − SM, J=3

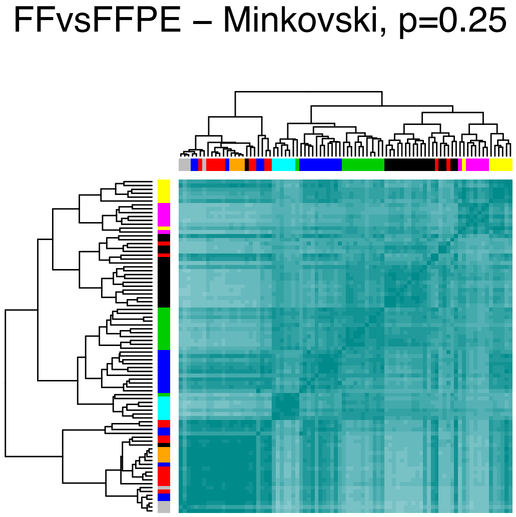
FFvsFFPE – Minkovski, p=0.25

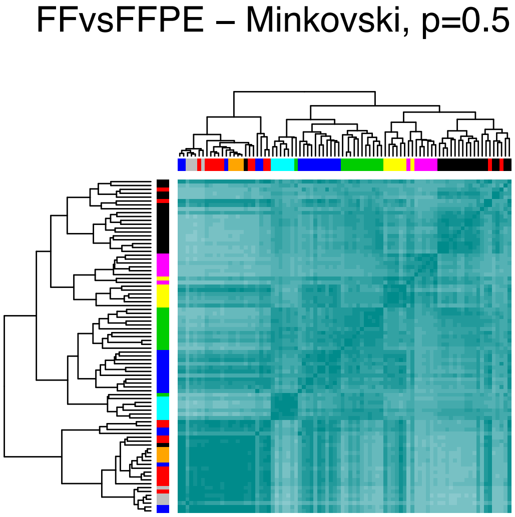
FFvsFFPE – Minkovski, p=0.5

FFvsFFPE – Minkovski, p=0.75

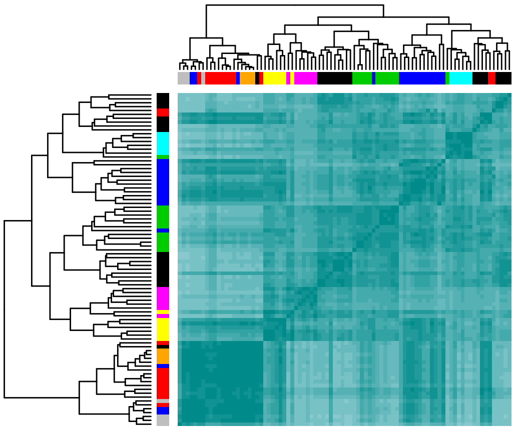

FFvsFFPE – Minkovski, p=3

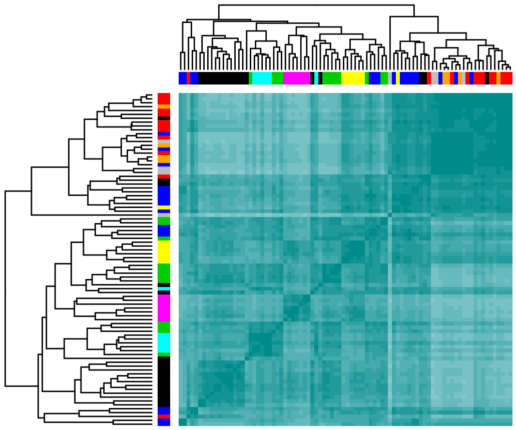

FFvsFFPE – Minkovski, p=4

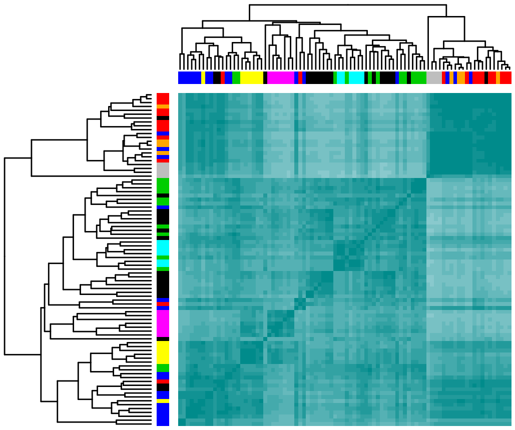

FFvsFFPE – Minkovski, p=5

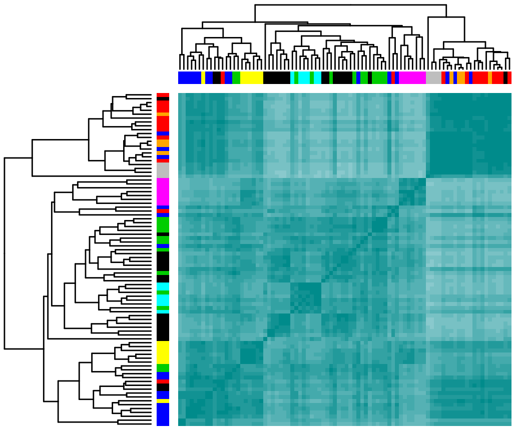

FFvsFFPE – Manhattan

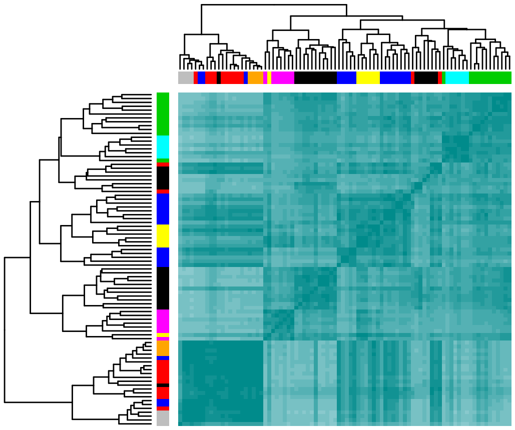

FFvsFFPE – Pearson

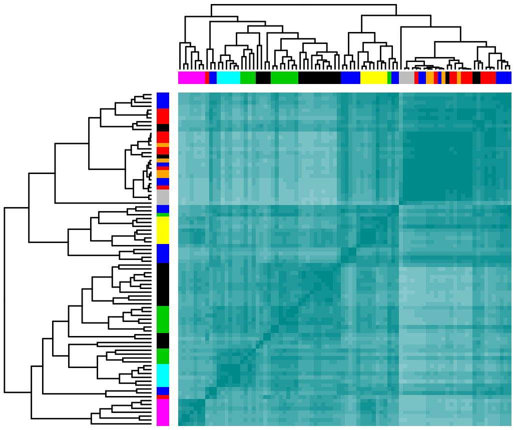

Supplement: Supplementary file 1 — Supplementary Information. [file 41598_2021_678_MOESM1_ESM.pdf]
